# Supplementary material for: An Aminopyrimidone and Aminoimidazoles Alkaloids from the Rodrigues Calcareous Marine Sponge Ernsta naturalis
Source: Mar Drugs. 2022 Oct 13;20(10):637. doi: 10.3390/md20100637 (PMC9604632; doi:10.3390/md20100637)

Supporting information

An Aminopyrimidone and Aminoimidazoles Alkaloids from the Rodrigues Calcareous Marine Sponge *Ernsta naturalis*

Pierre-Eric Campos[1,2], Gaëtan Herbette[3], Laetitia Fougère[2], Patricia Clerc[1], Florent Tintillier[1], Nicole J. de Voogd[4,5], Géraldine Le Goff[6], Jamal Ouazzani[6] and Anne Gauvin-Bialecki[1*]

[1] Laboratoire de chimie et de biotechnologie des produits naturels, Faculté des Sciences et Technologies, Université de La Réunion, 15 Avenue René Cassin, CS 92003, 97744 Saint-Denis Cedex 9, La Réunion, France
[2] Institut de Chimie Organique et Analytique, Université d'Orléans – CNRS - Pôle de chimie, rue de Chartres – UMR 6759, BP6759, 45067 Orléans Cedex 2, France
[3] Aix-Marseille Univ, CNRS, Centrale Marseille, FSCM, Spectropole, Campus de St Jérôme-Service 511, 13397 Marseille, France
[4] Naturalis Biodiversity Center, Darwinweg 2, 2333 CR Leiden, Netherlands
[5] Institute of Environmental Sciences, Leiden University, Einsteinweg 2, 2333 CC Leiden, Netherlands
[6] Institut de Chimie des Substances Naturelles, CNRS UPR 2301, Univ. Paris-Sud, Université Paris-Saclay, 1, av. de la Terrasse, 91198 Gif-sur-Yvette, France
* Correspondence: anne.bialecki@univ-reunion.fr; Tel.: +262 262 93 81 97

Supporting information

Figure S1: HRESIMS spectrum for ernstine A (1) 4
Figure S2: ^{1}H NMR (600 MHz, CD_3OD) spectrum for ernstine A (1) 4
Figure S3: ^{1}H-^{1}H COSY NMR (600 MHz) spectrum for ernstine A (1) 5
Figure S4: ^{1}H-^{13}C HSQC NMR (600 MHz) spectrum for ernstine A (1) 5
Figure S5: ^{1}H-^{13}C HMBC NMR (600 MHz) spectrum for ernstine A (1) 6
Figure S6: ^{1}H-^{1}H NOESY NMR (600 MHz) spectrum for ernstine A (1) 7
Figure S7: HRESIMS spectrum for phorbatopsin D (2) 7
Figure S8: ^{1}H NMR (600 MHz, CD_3OD) spectrum for phorbatopsin D (2) 8
Figure S9: ^{13}C NMR (125 MHz, CD_3OD) spectrum for phorbatopsin D (2) 8
Figure S10: ^{1}H-^{13}C HSQC NMR (600 MHz) spectrum for phorbatopsin D (2) 9
Figure S11: ^{1}H-^{13}C HMBC NMR (600 MHz) spectrum for phorbatopsin D (2) 9
Figure S12: HRESIMS spectrum for phorbatopsin E (3) 10
Figure S13: ^{1}H NMR (600 MHz, CD_3OD) spectrum for phorbatopsin E (3) 10
Figure S14: ^{1}H-^{13}C HSQC NMR (600 MHz) spectrum for phorbatopsin E (3) 11
Figure S15: ^{1}H-^{13}C HMBC NMR (600 MHz) spectrum for phorbatopsin E (3) 11
Figure S16: ^{1}H-^{1}H NOESY NMR (600 MHz) spectrum for phorbatopsin E (3) 12
Figure S17: HRESIMS spectrum for calcaridine C (4) 12
Figure S18: ^{1}H NMR (600 MHz, CD_3OD) spectrum for calcaridine C (4) 13
Figure S19: ^{13}C NMR (125 MHz, CD_3OD) spectrum for calcaridine C (4) 14
Figure S20: ^{1}H-^{1}H COSY NMR (600 MHz) spectrum for calcaridine C (4) 14
Figure S21: ^{1}H-^{13}C HSQC NMR (600 MHz) spectrum for calcaridine C (4) 15
Figure S22: ^{1}H-^{13}C HMBC NMR (600 MHz) spectrum for calcaridine C (4) 15
Figure S23: ^{1}H-^{1}H NOESY NMR (600 MHz) spectrum for calcaridine C (4) 16
Figure S24: HRESIMS spectrum for naamine H (5) 16
Figure S25: ^{1}H NMR (600 MHz, CD_3OD) spectrum for naamine H (5) 17
Figure S26: ^{1}H-^{13}C HSQC NMR (600 MHz) spectrum for naamine H (5) 18
Figure S27: ^{1}H-^{13}C HMBC NMR (600 MHz) spectrum for naamine H (5) 18
Figure S28: ^{1}H-^{1}H NOESY NMR (600 MHz) spectrum for naamine H (5) 19
Figure S29: HRESIMS spectrum for naamidine J (6) 19
Figure S30: ^{1}H NMR (600 MHz, CD_3OD) spectrum for naamidine J (6) 20
Figure S31: ^{13}C NMR (125 MHz, CD_3OD) spectrum for naamidine J (6) 20
Figure S32: ^{1}H-^{1}H COSY NMR (600 MHz) spectrum for naamidine J (6) 21
Figure S33: ^{1}H-^{13}C HSQC NMR (600 MHz) spectrum for naamidine J (6) 21
Figure S34: ^{1}H-^{13}C HMBC NMR (600 MHz) spectrum for naamidine J (6) 22
Figure S35: ^{1}H-^{1}H NOESY NMR (600 MHz) spectrum for naamidine J (6) 22
Figure S36: HRESIMS spectrum for naamine I (7) 23
Figure S37: HRESIMS spectrum for naamidine K (8) 23
Figure S38: ^{1}H NMR (600 MHz, CD_3OD) spectrum for mixture of naamine I (7) and naamidine K (8) 24
Figure S39: ^{1}H-^{13}C HSQC NMR (600 MHz) spectrum for mixture of naamine I (7) and naamidine K (8) 24
Figure S40: ^{1}H-^{13}C HMBC NMR (600 MHz) spectrum for mixture of naamine I (7) and naamidine K (8) 25
Figure S41: ^{1}H NMR (600 MHz, CD_3OD) spectrum for thymidine (9) 25
Figure S42: ^{13}C NMR (125 MHz, CD_3OD) spectrum for thymidine (9) 26
Figure S43: ^{1}H-^{1}H COSY NMR (600 MHz) spectrum for thymidine (9) 26
Figure S44: ^{1}H-^{13}C HSQC NMR (600 MHz) spectrum for thymidine (9) 27
Figure S45: ^{1}H-^{13}C HMBC NMR (600 MHz) spectrum for thymidine (9) 27
Figure S46: MS/MS spectra of the isolated compounds were deposited in the GNPS spectral libraries under following identifier 28
Figure S47: MS/MS spectrum of ernstine A (1) 28
Figure S48: MS/MS spectrum of Phorbatopsin D (2) 28

Figure S49: MS/MS spectrum of Phorbatopsin E(3) 29
Figure S50: MS/MS spectrum of Naamine H (5) 29
Figure S51: MS/MS spectrum of Naamine I (7) 29
Figure S52: MS/MS spectrum of Naamidine J (6) 30
Figure S53: MS/MS spectrum of Naamidine K (8) 30
Figure S54: MS/MS spectrum of Calcaridine C (4) 30

In situ Ernsta naturalis Photo

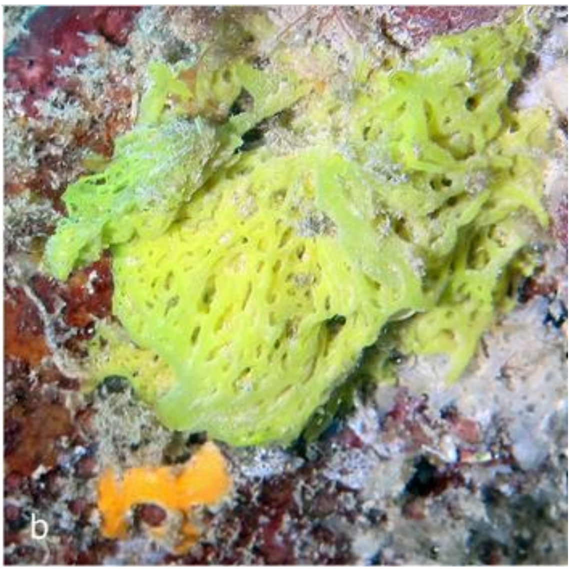

Figure S1: HRESIMS spectrum for ernstine A (1)

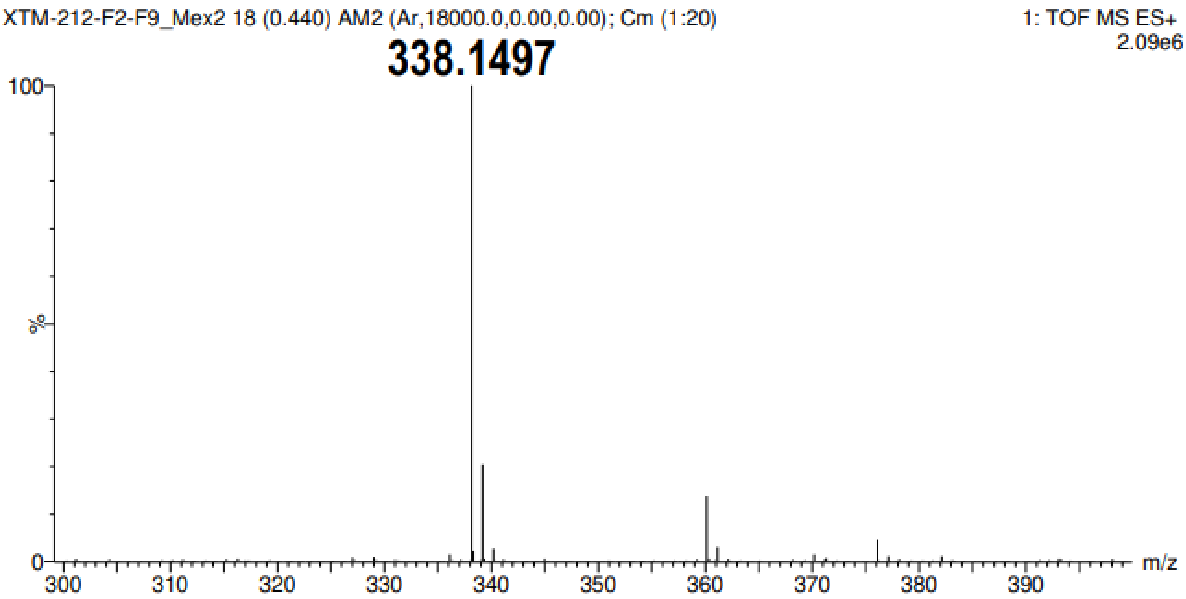

Figure S2: ^{1}H NMR (600 MHz, CD_3OD) spectrum for ernstine A (1)

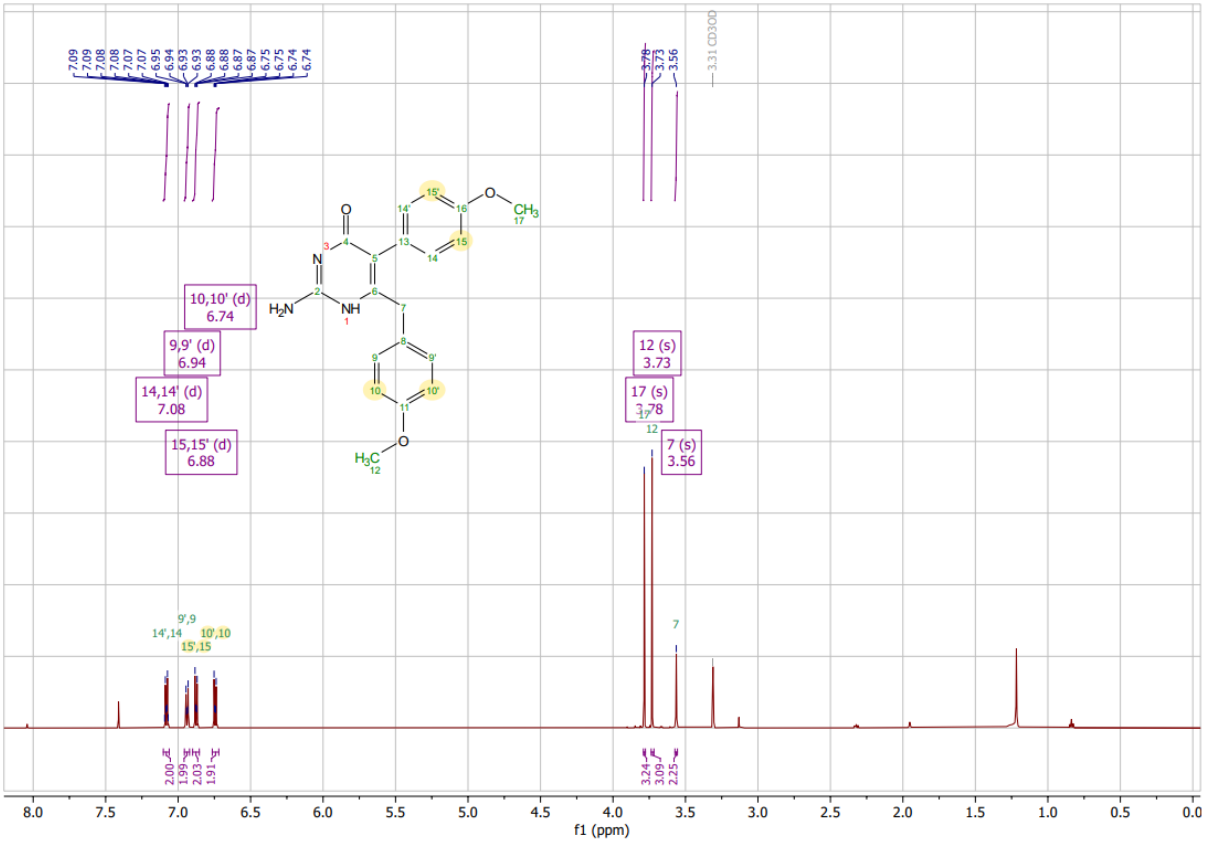

Figure S3: ^{1}H-^{1}H COSY NMR (600 MHz) spectrum for ernstine A (1)

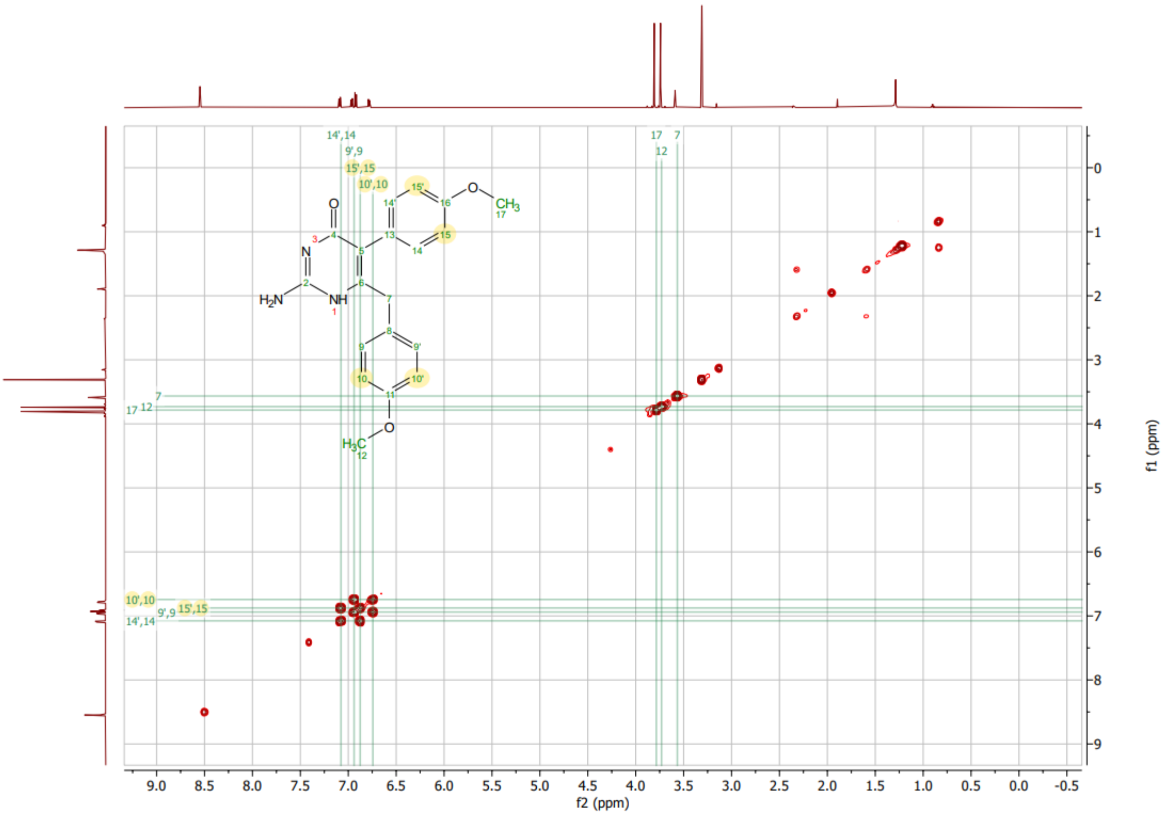

Figure S4: ^{1}H-^{13}C HSQC NMR (600 MHz) spectrum for ernstine A (1)

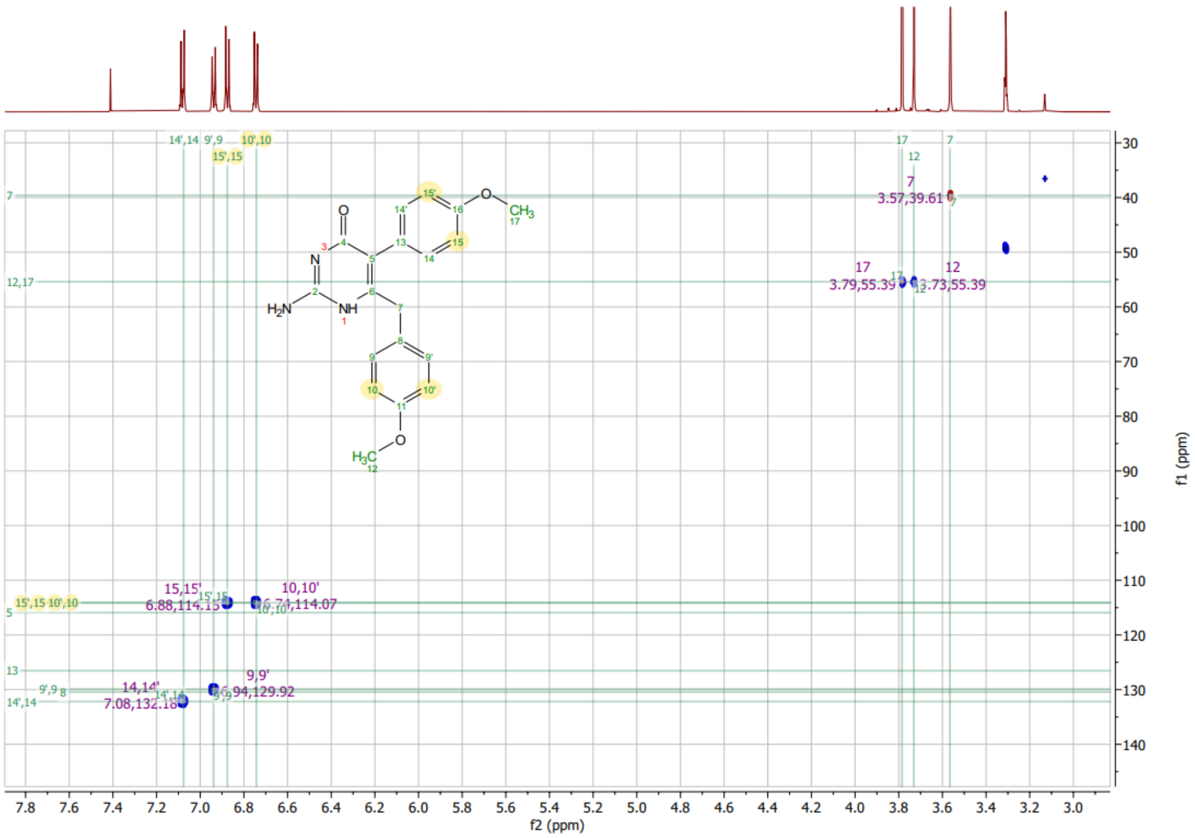

Figure S5: ^{1}H-^{13}C HMBC NMR (600 MHz) spectrum for ernstine A (1)

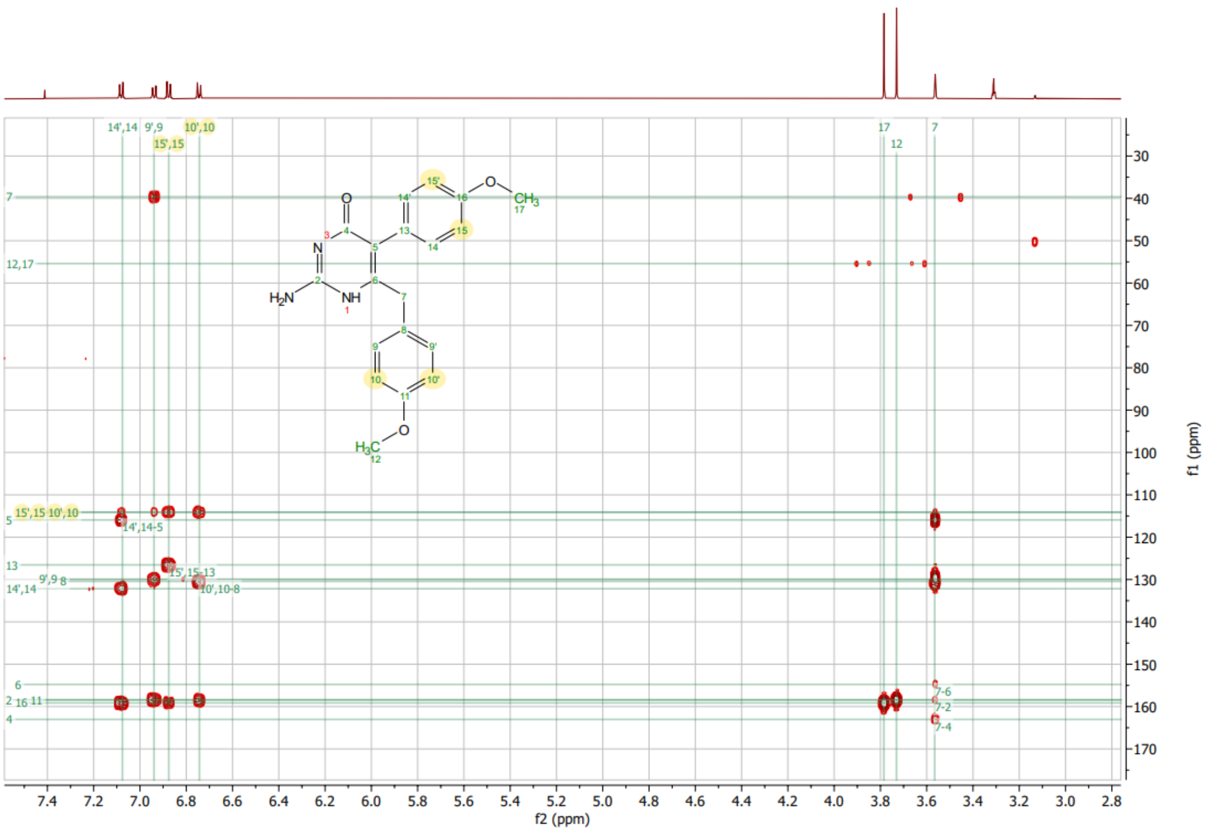

Figure S6: ^{1}H-^{1}H NOESY NMR (600 MHz) spectrum for ernstine A (1)

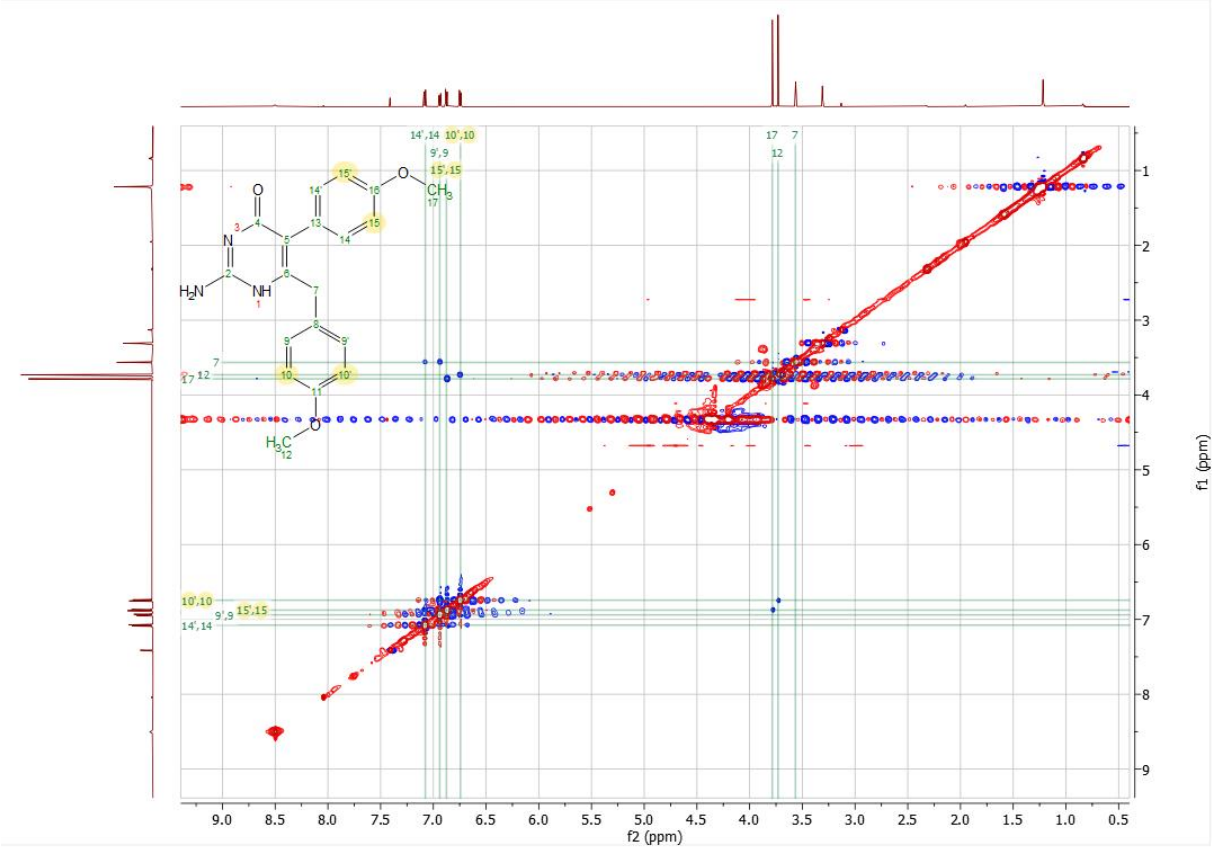

Figure S7: HRESIMS spectrum for phorbatopsin D (2)

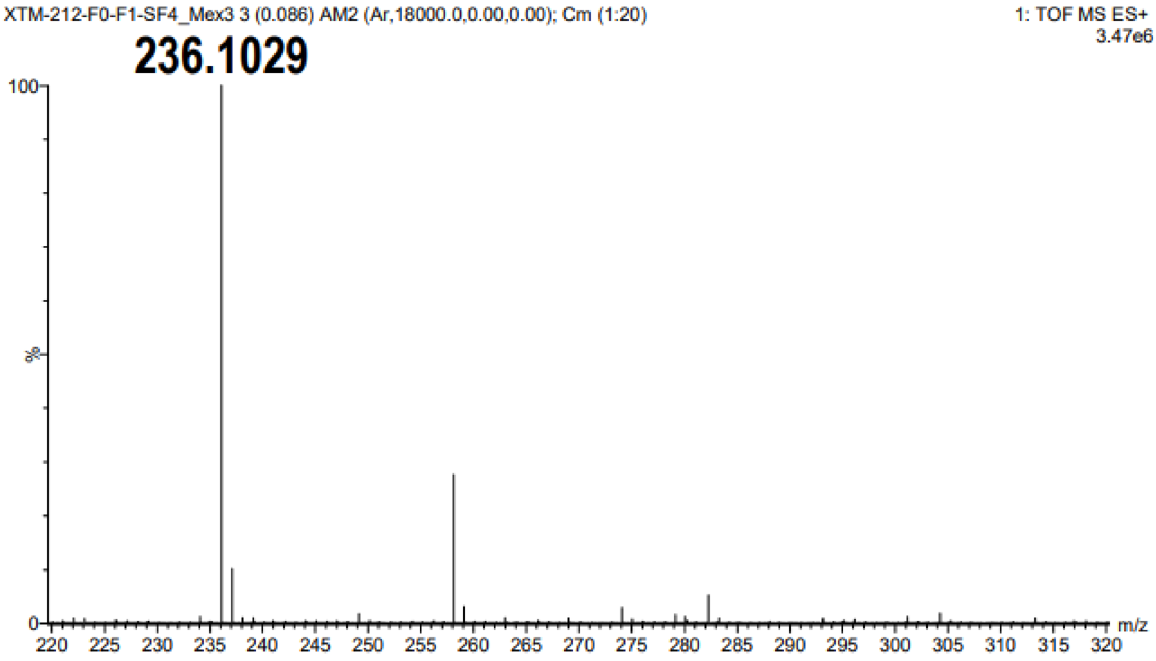

Figure S8: ^{1}H NMR (600 MHz, CD_3OD) spectrum for phorbatopsin D (2)

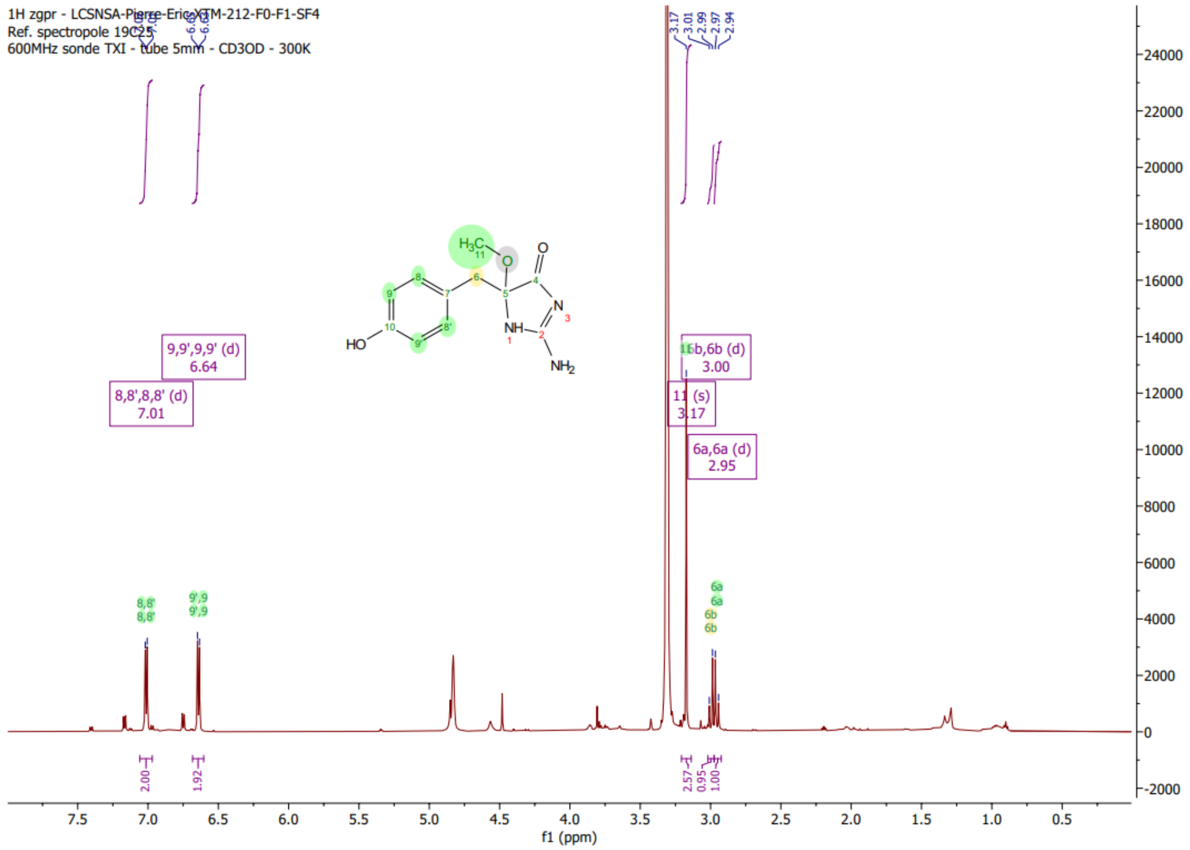

Figure S9: ^{13}C NMR (125 MHz, CD_3OD) spectrum for phorbatopsin D (2)

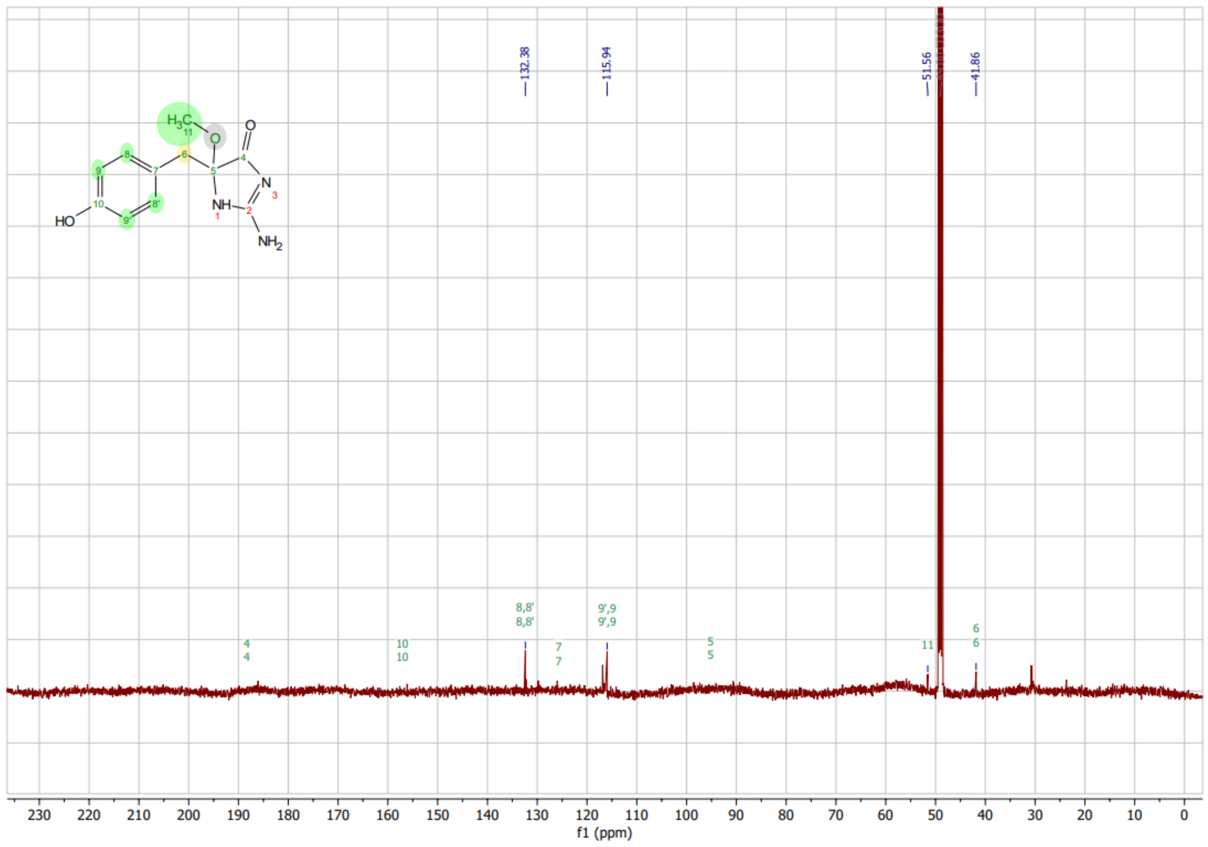

Figure S10: ^{1}H-^{13}C HSQC NMR (600 MHz) spectrum for phorbatopsin D (2)

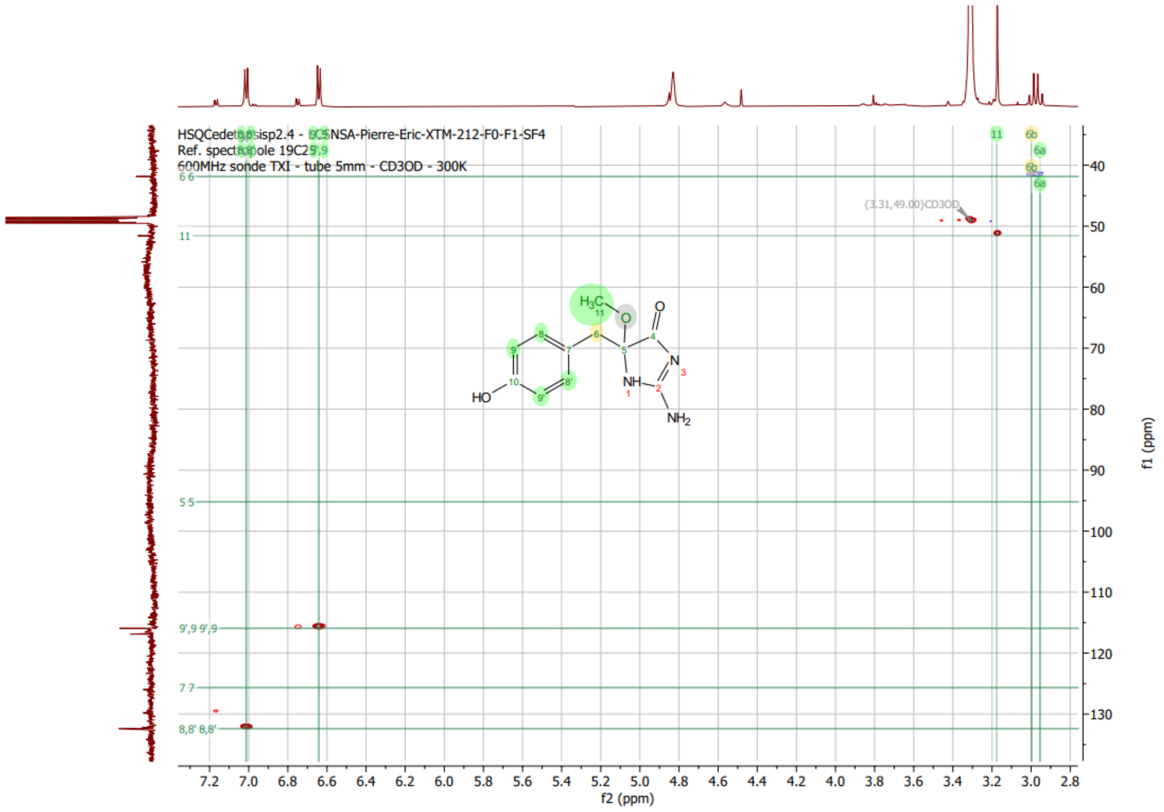

Figure S11: ^{1}H-^{13}C HMBC NMR (600 MHz) spectrum for phorbatopsin D (2)

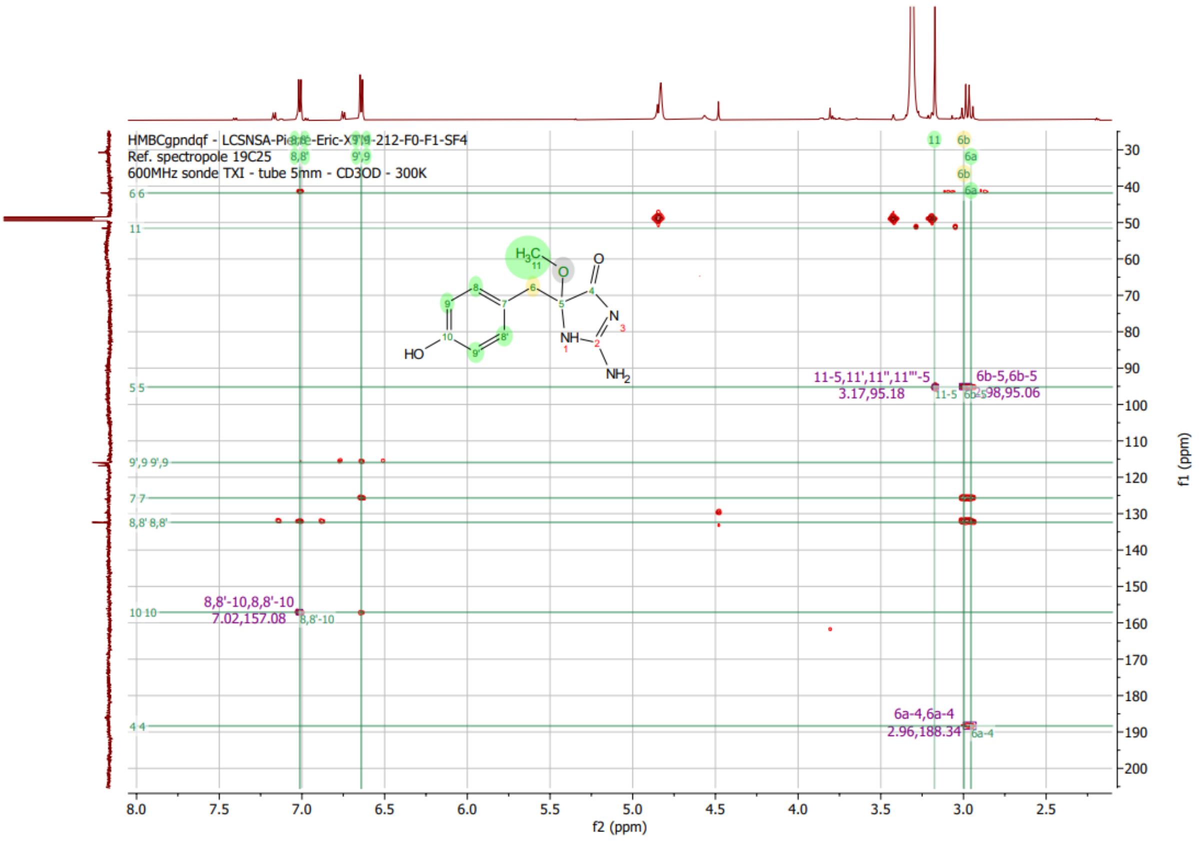

Figure S12: HRESIMS spectrum for phorbatopsin E (3)

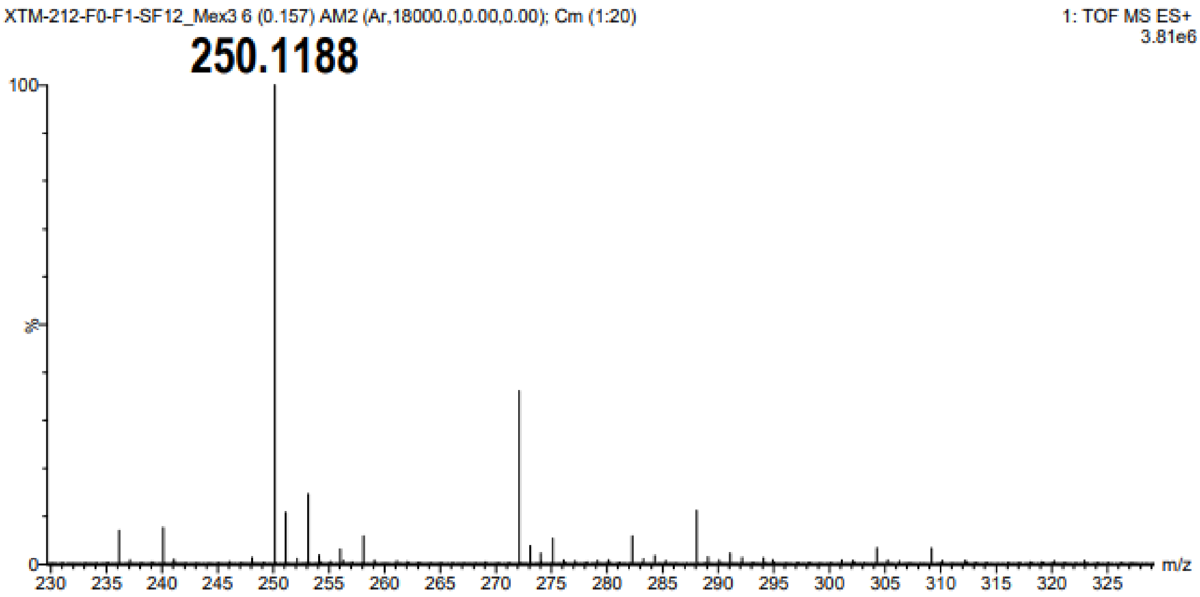

Figure S13: ^{1}H NMR (600 MHz, CD_3OD) spectrum for phorbatopsin E (3)

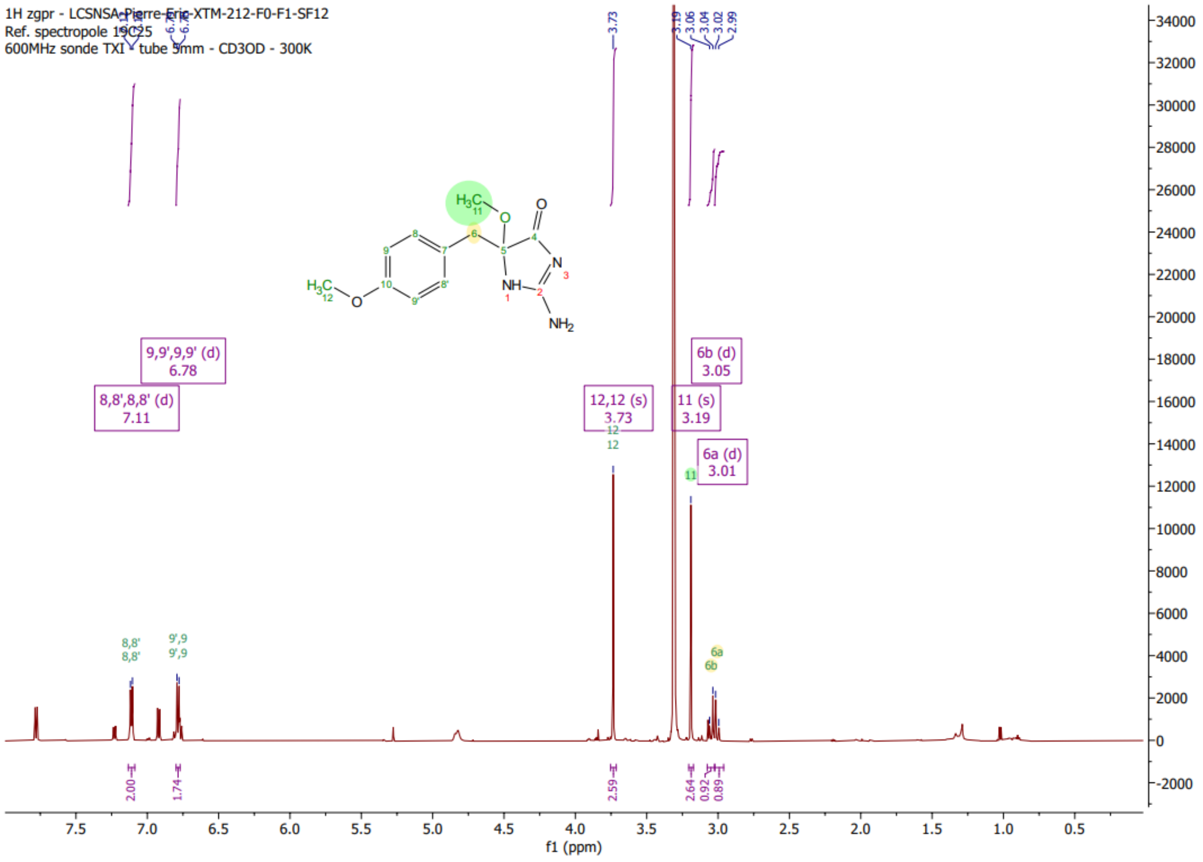

Figure S14: ^{1}H-^{13}C HSQC NMR (600 MHz) spectrum for phorbatopsin E (3)

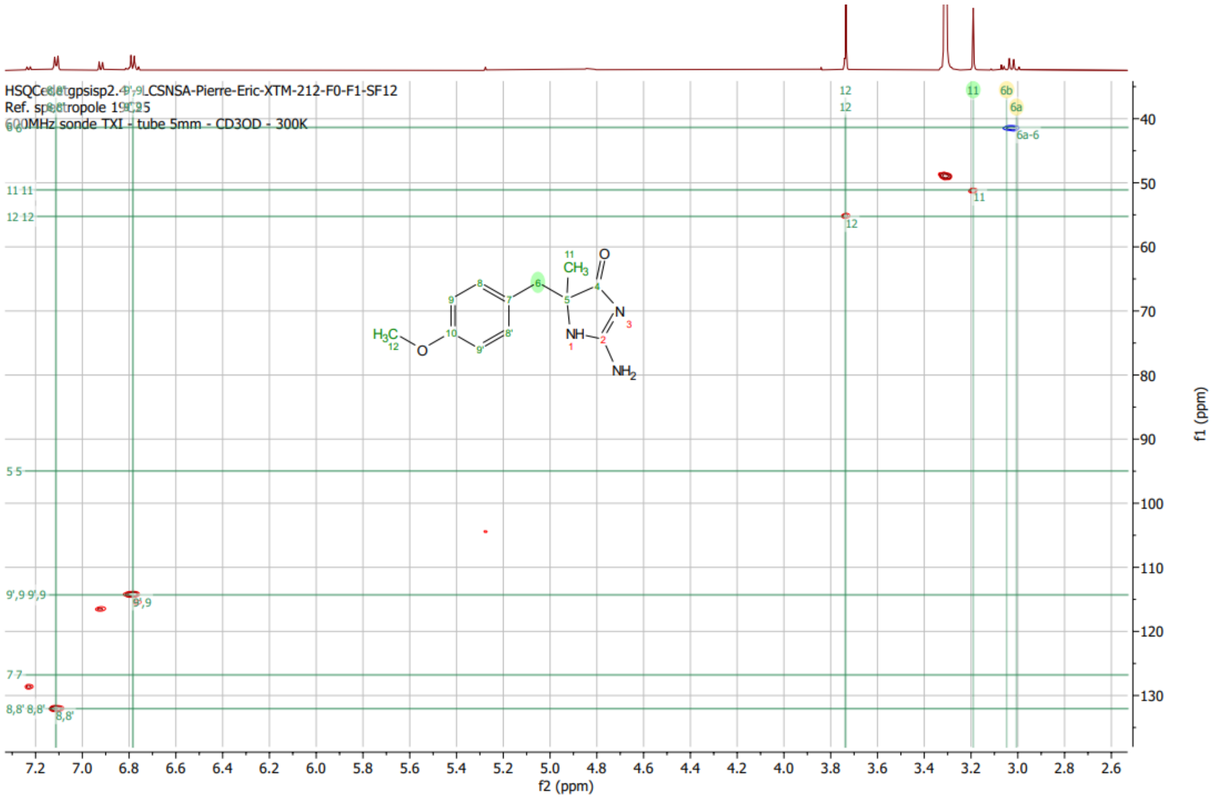

Figure S15: ^{1}H-^{13}C HMBC NMR (600 MHz) spectrum for phorbatopsin E (3)

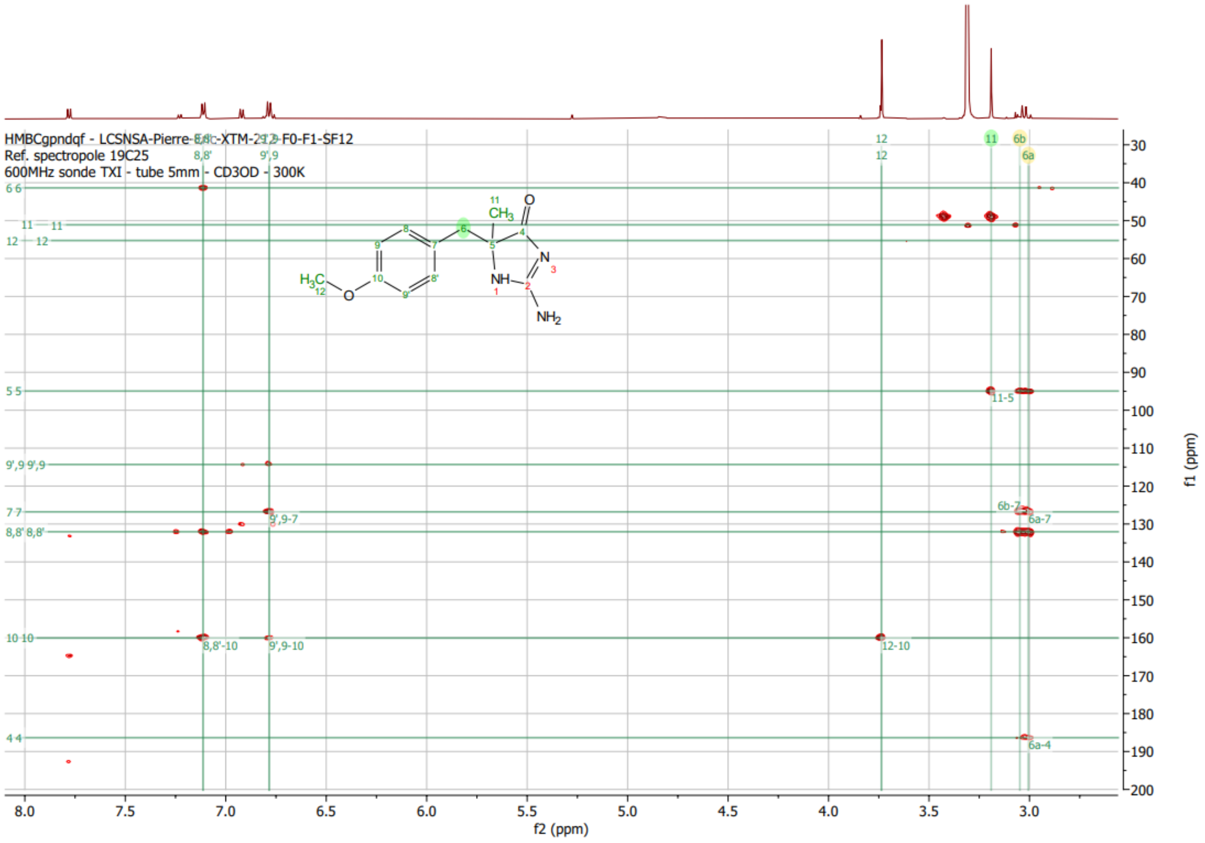

Figure S16: ^{1}H-^{1}H NOESY NMR (600 MHz) spectrum for phorbatopsin E (3)

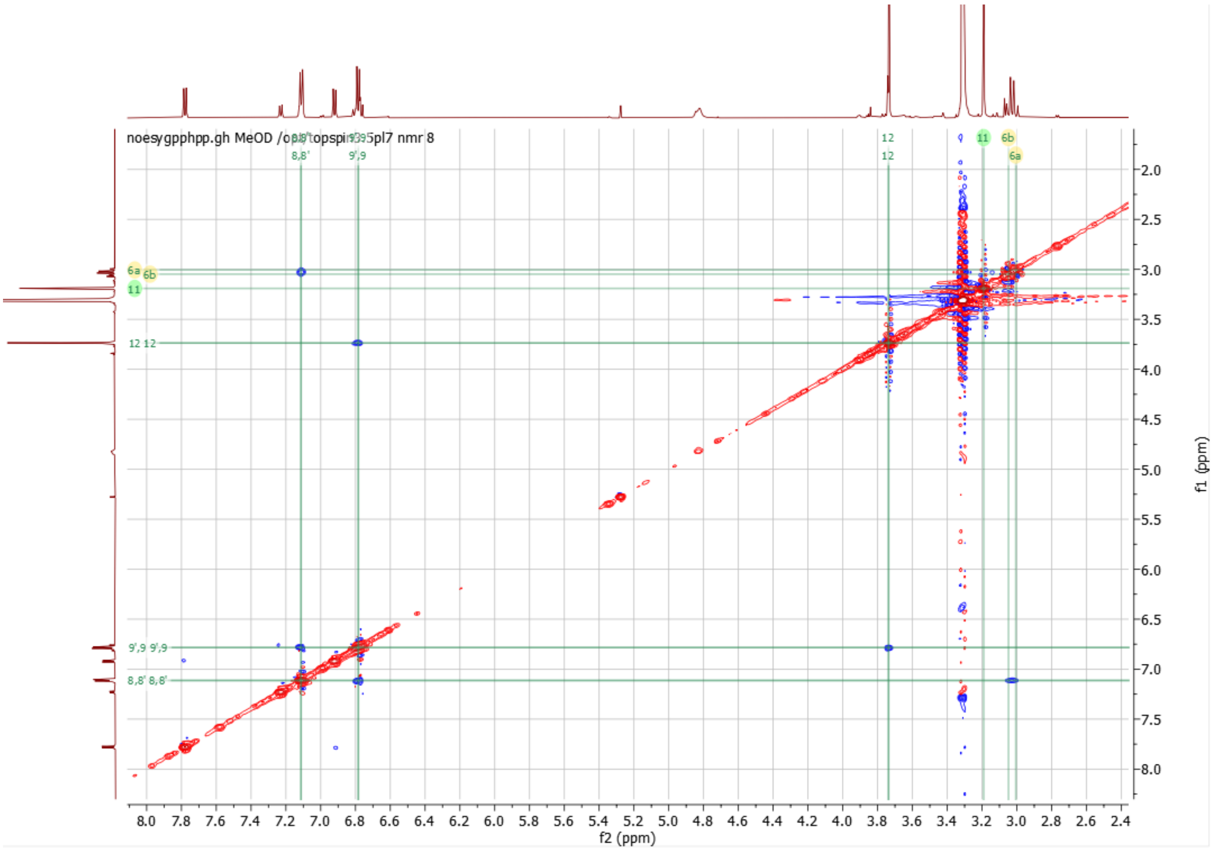

Figure S17: HRESIMS spectrum for calcaridine C (4)

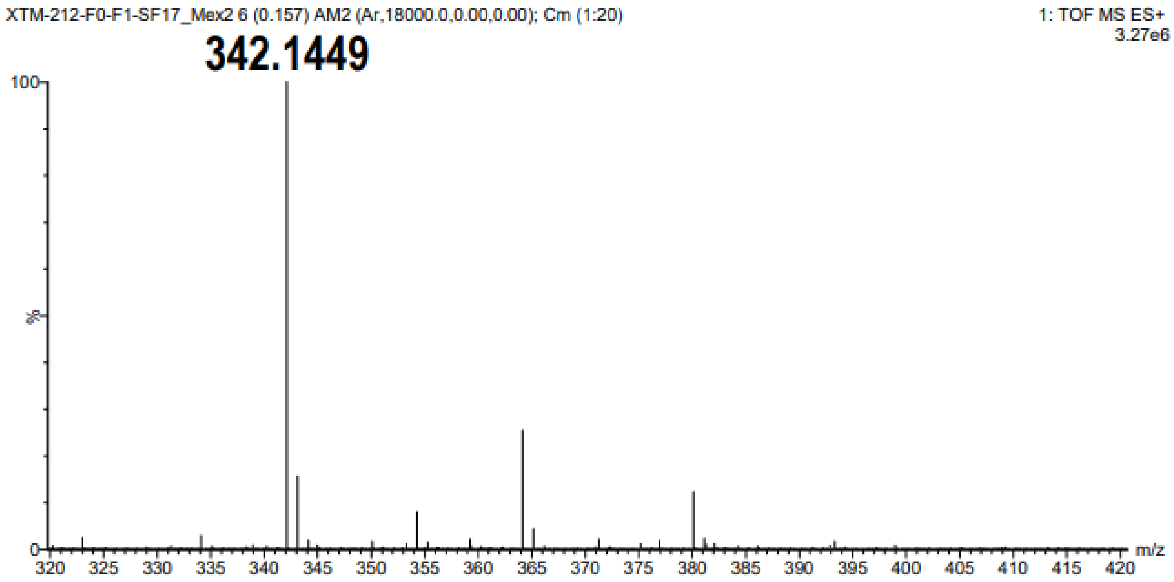

Figure S18: ^{1}H NMR (600 MHz, CD_3OD) spectrum for calcaridine C (4)

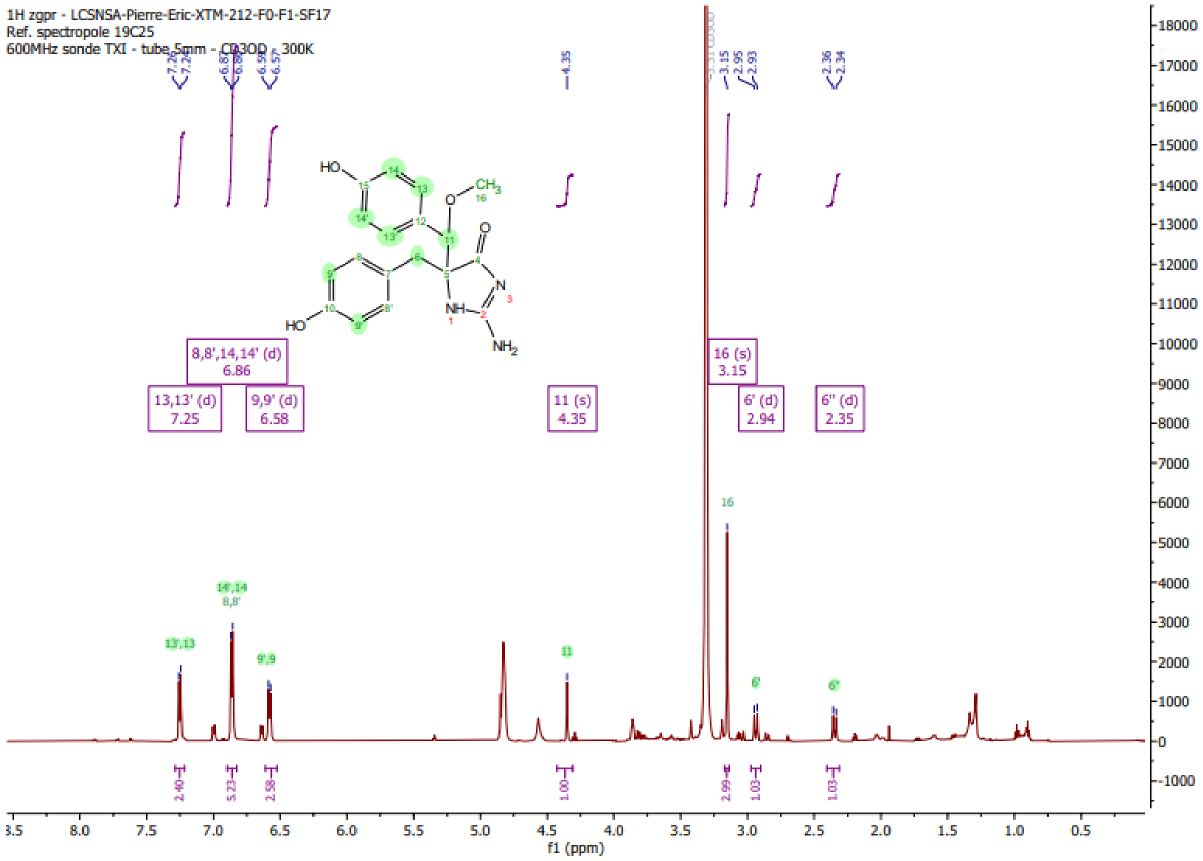

Figure S19: ^{13}C NMR (125 MHz, CD_3OD) spectrum for calcaridine C (4)

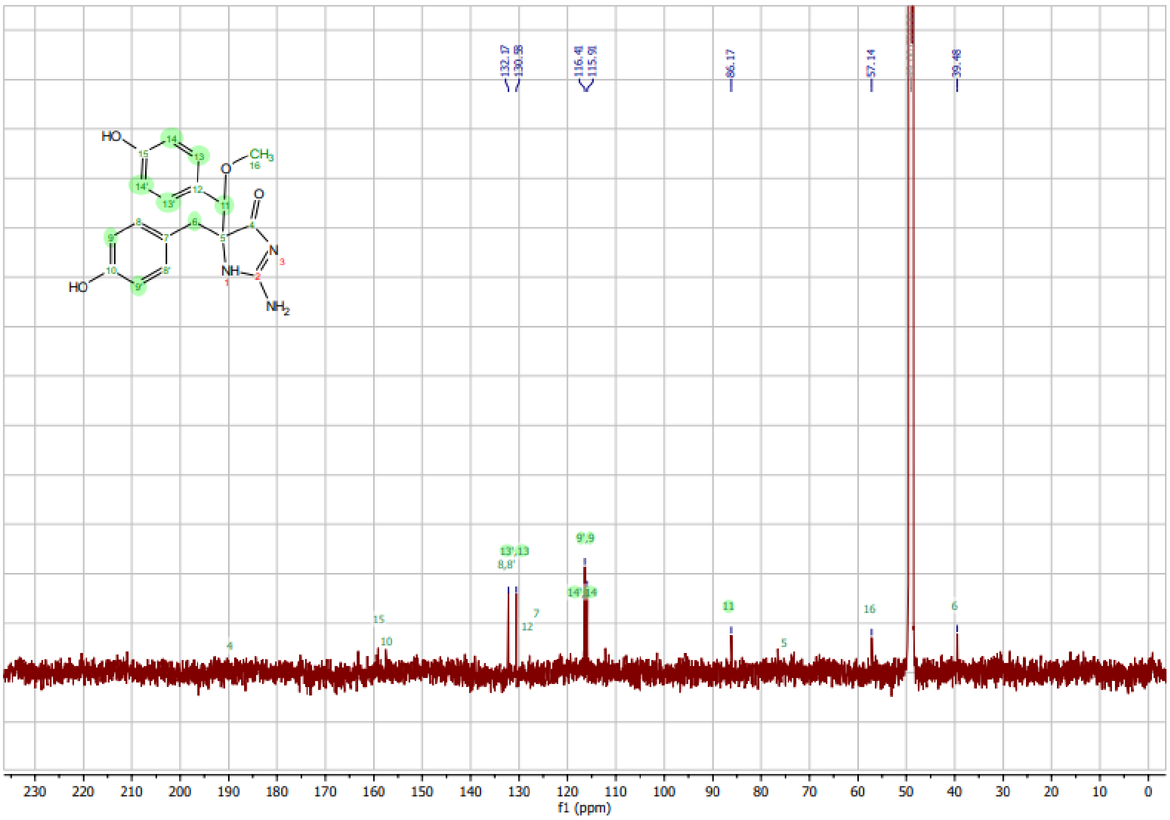

Figure S20: ^{1}H-^{1}H COSY NMR (600 MHz) spectrum for calcaridine C (4)

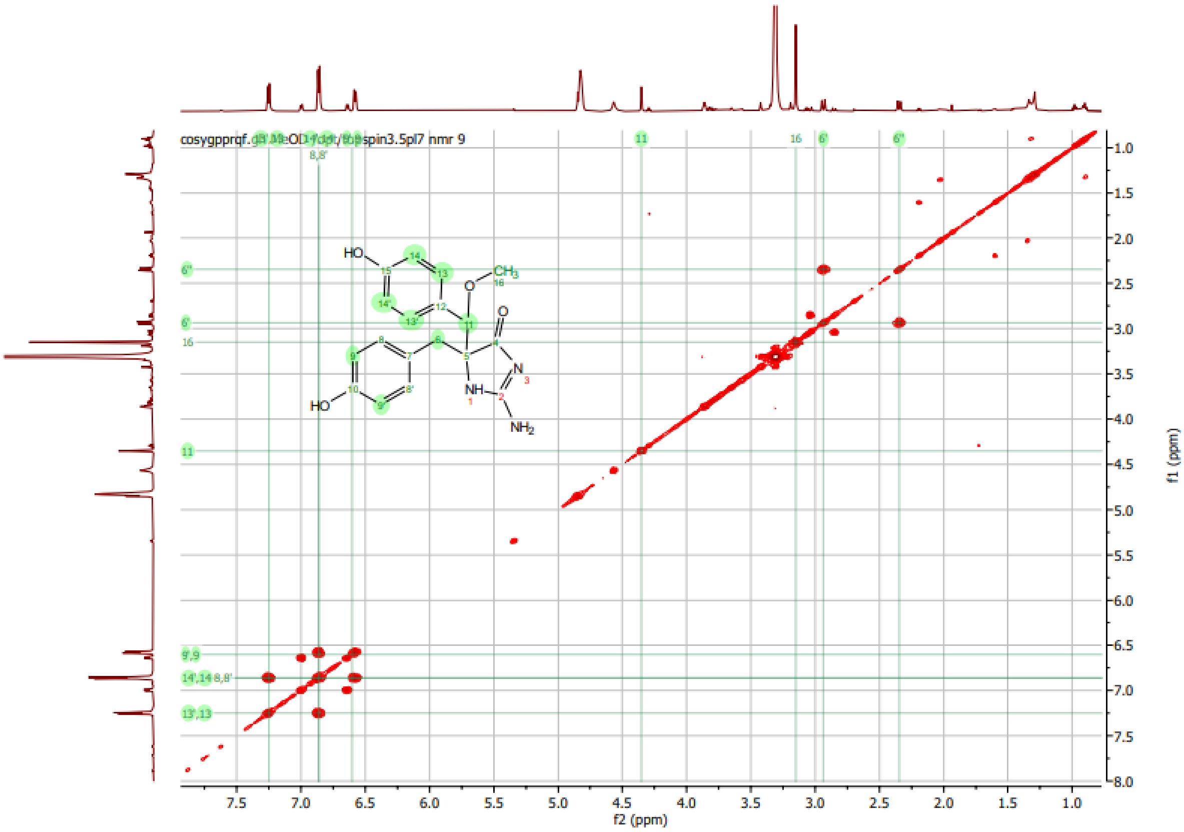

Figure S21: ^{1}H-^{13}C HSQC NMR (600 MHz) spectrum for calcaridine C (4)

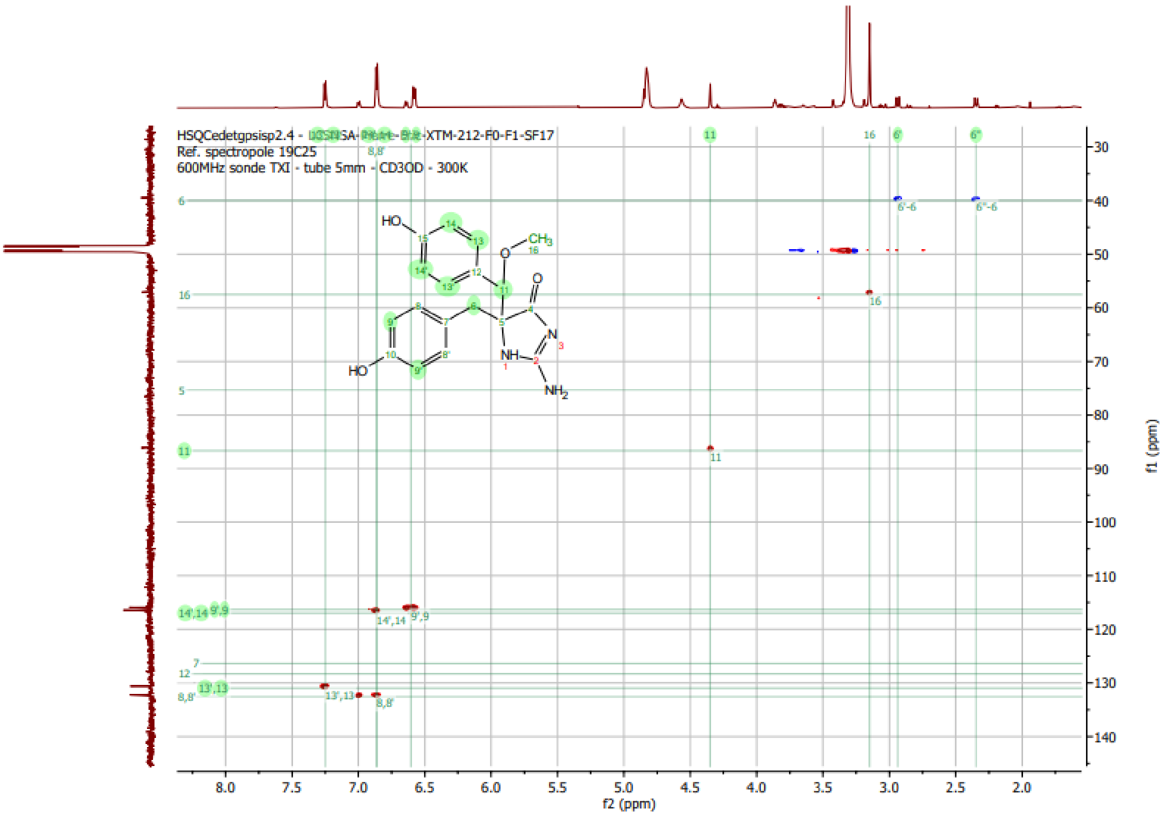

Figure S22: ^{1}H-^{13}C HMBC NMR (600 MHz) spectrum for calcaridine C (4)

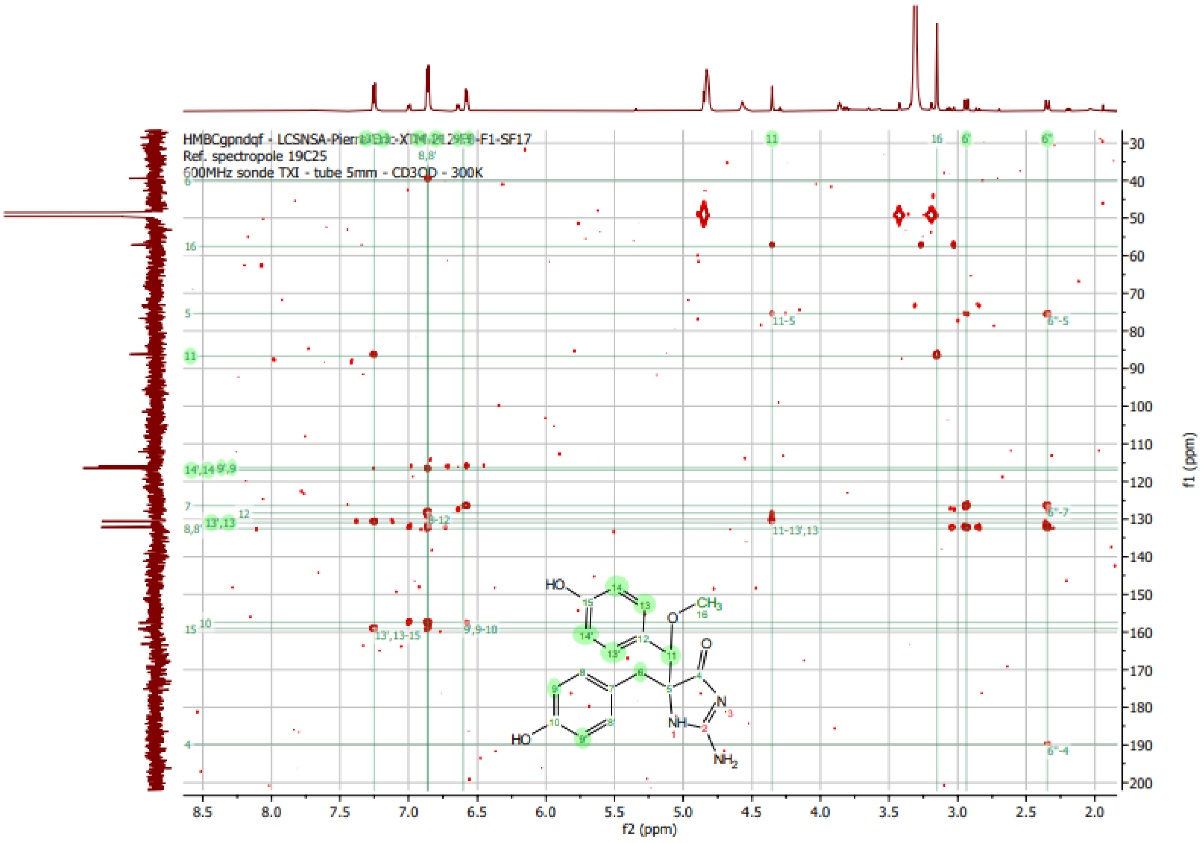

Figure S23: ^{1}H-^{1}H NOESY NMR (600 MHz) spectrum for calcaridine C (4)

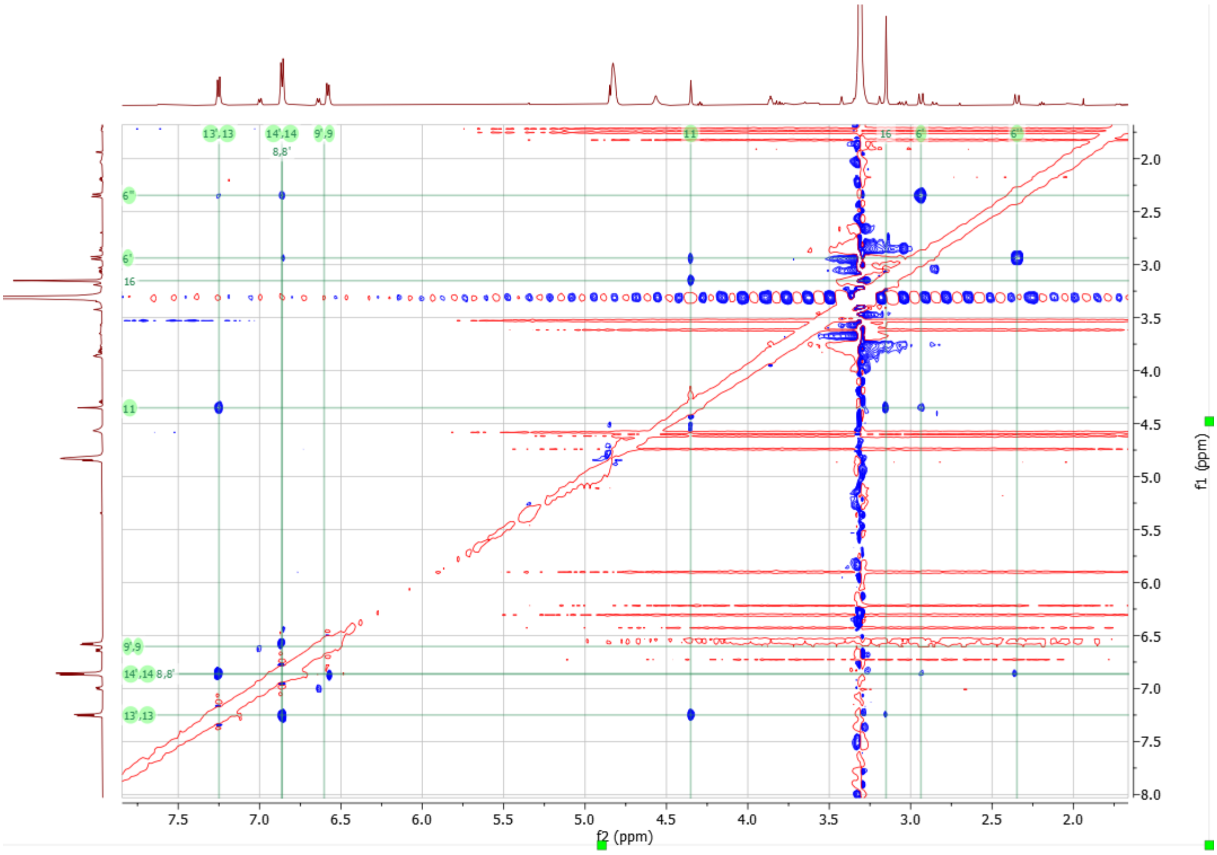

Figure S24: HRESIMS spectrum for naamine H (5)

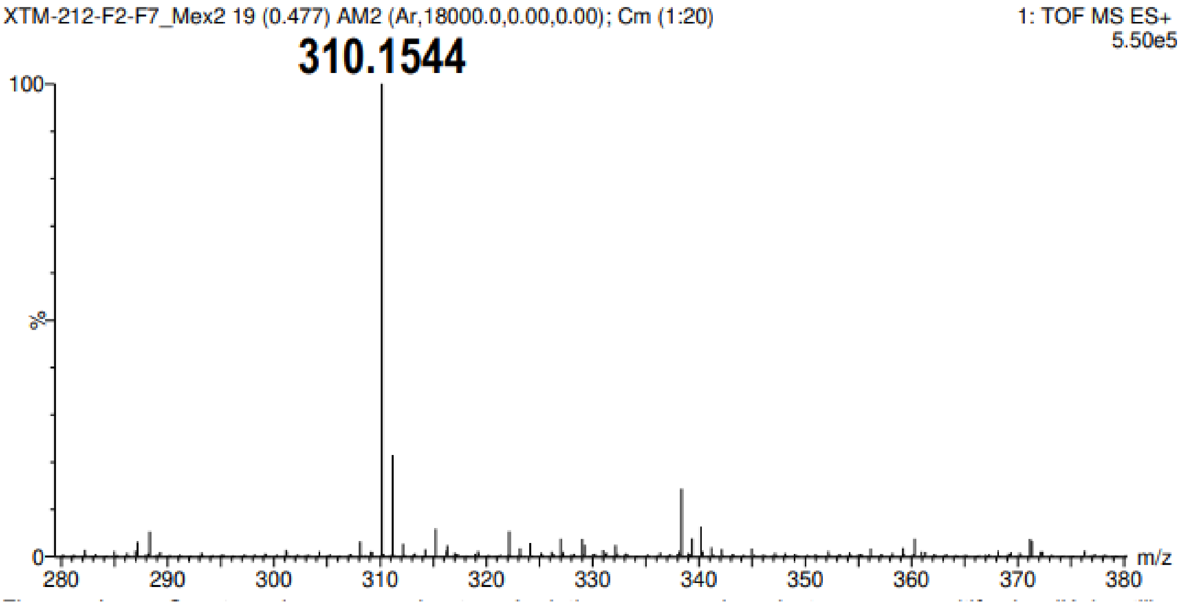

Figure S25: ^{1}H NMR (600 MHz, CD_3OD) spectrum for naamine H (5)

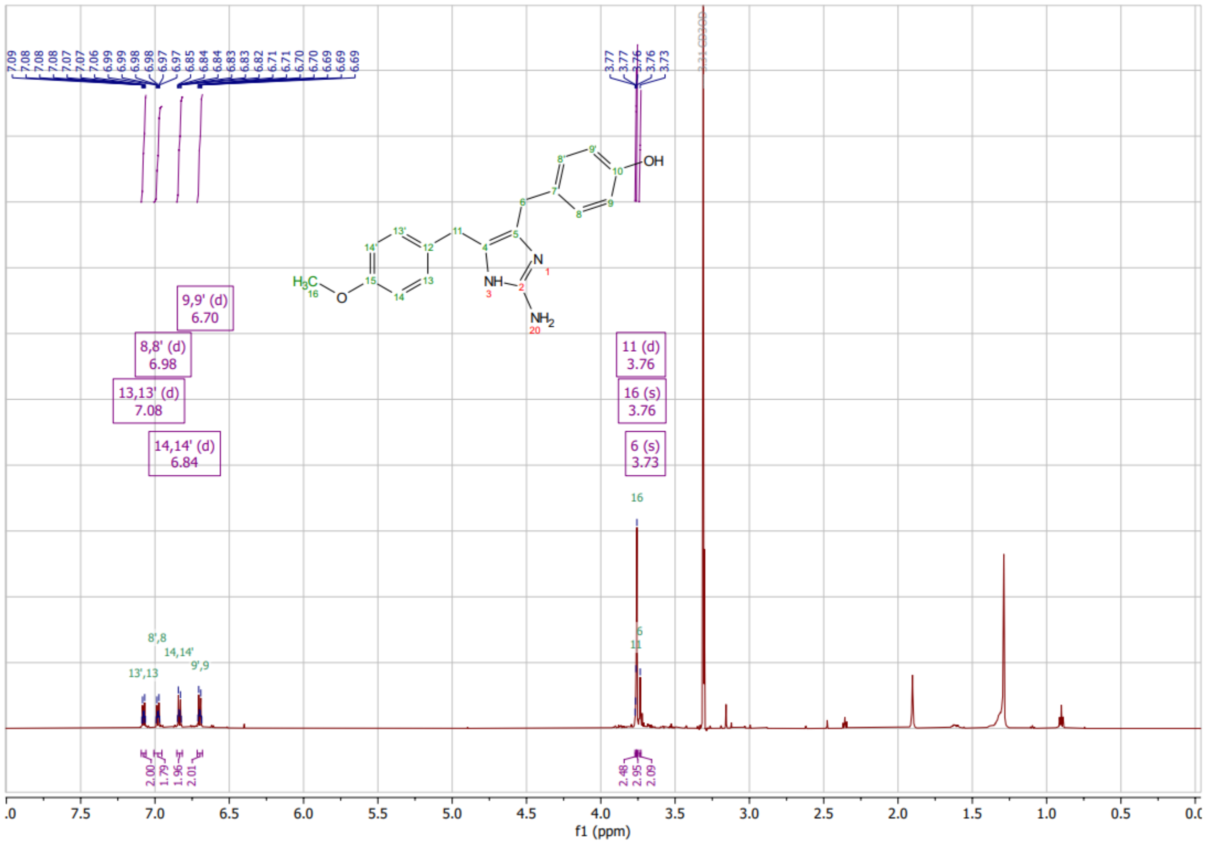

Figure S26: ^{1}H-^{13}C HSQC NMR (600 MHz) spectrum for naamine H (5)

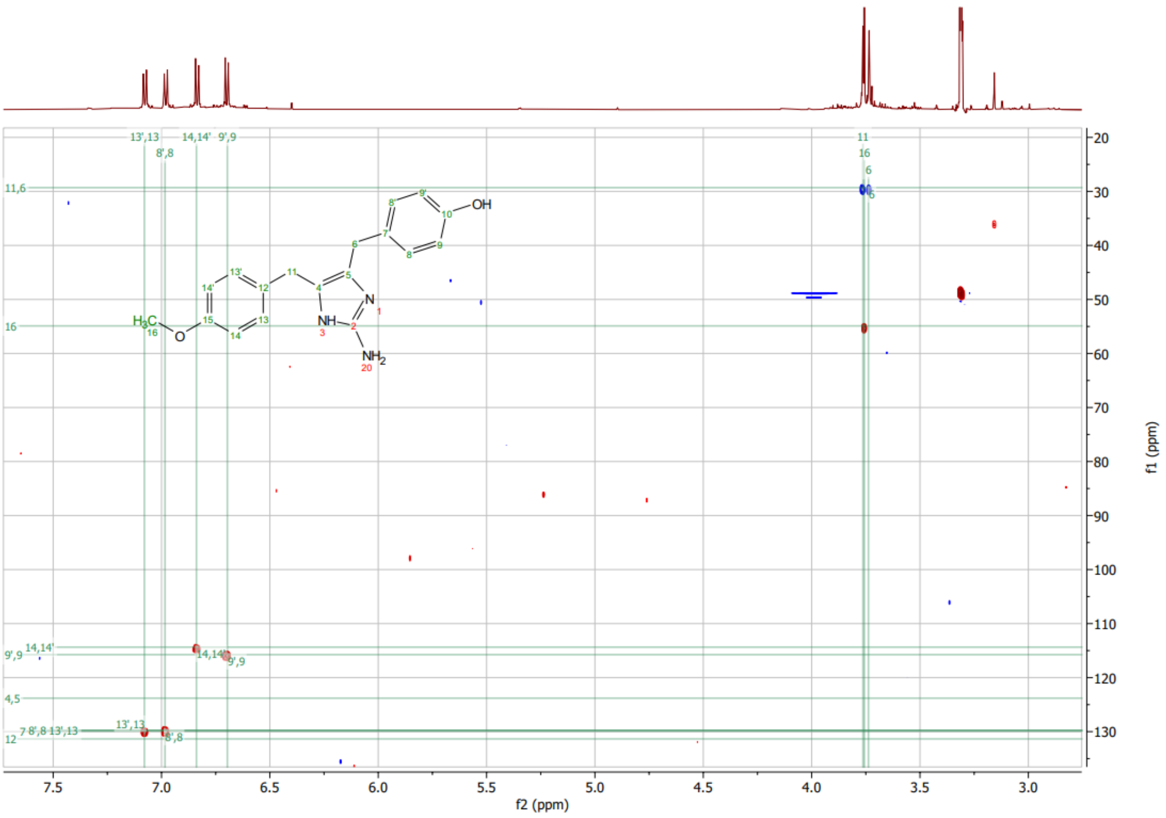

Figure S27: ^{1}H-^{13}C HMBC NMR (600 MHz) spectrum for naamine H (5)

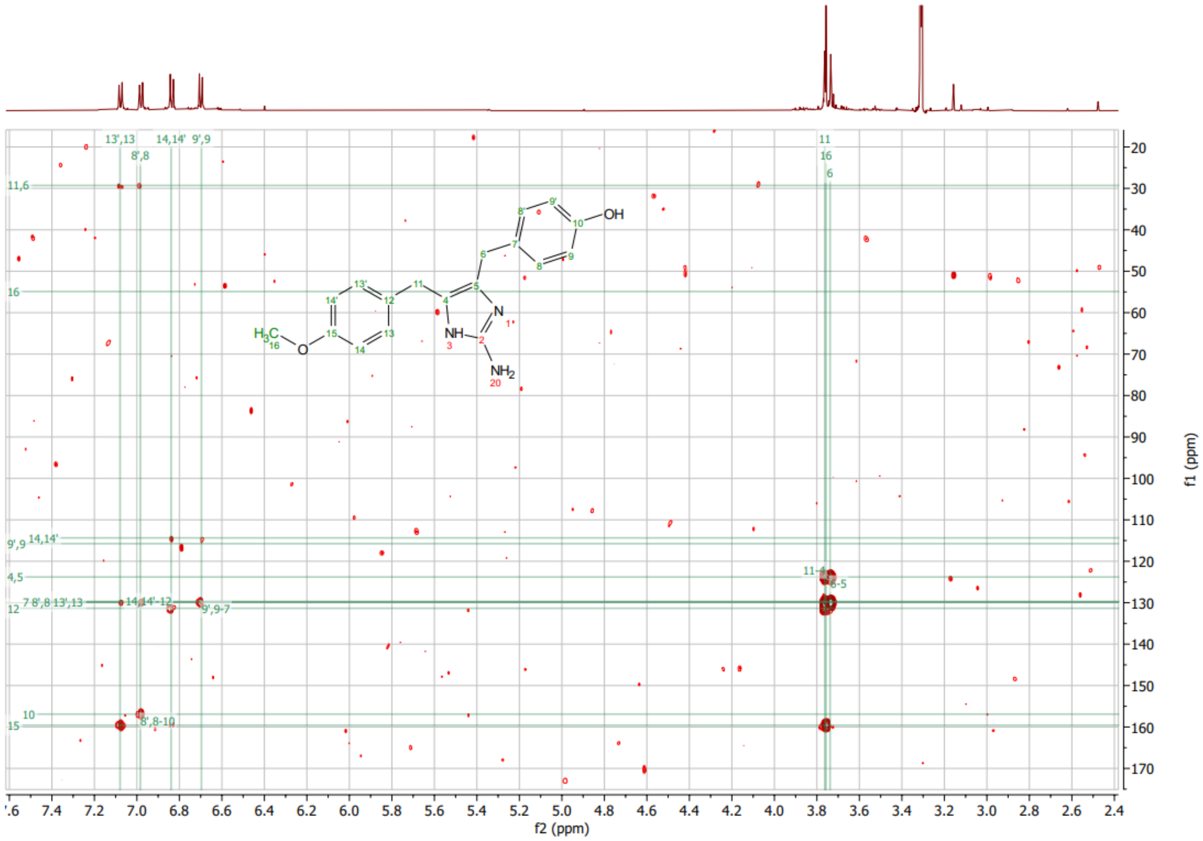

Figure S28: ^{1}H-^{1}H NOESY NMR (600 MHz) spectrum for naamine H (5)

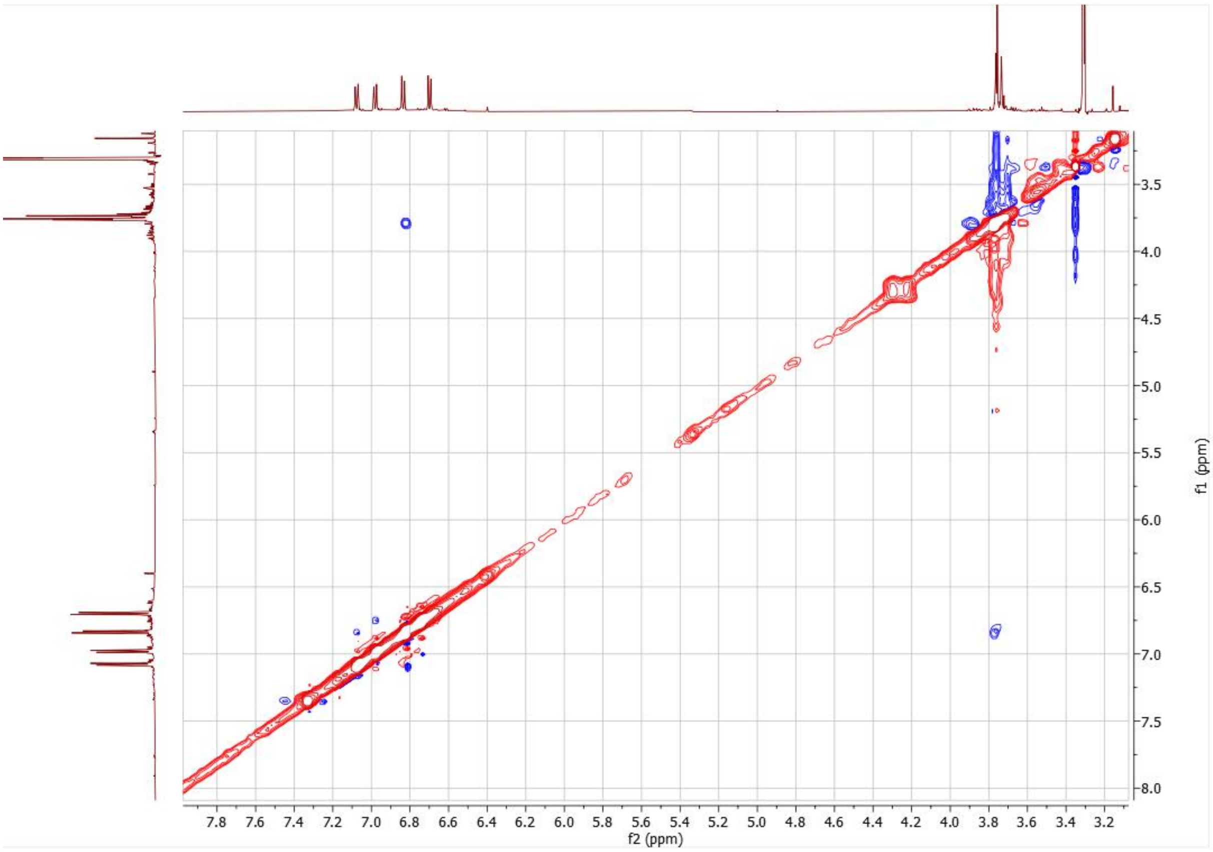

Figure S29: HRESIMS spectrum for naamidine J (6)

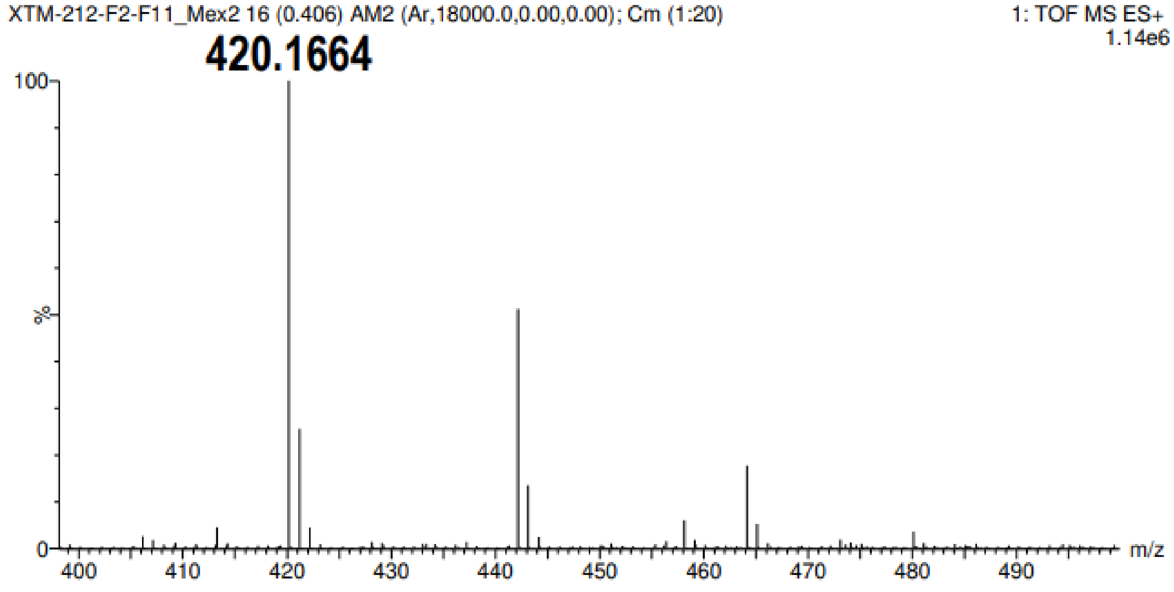

Figure S30: ^{1}H NMR (600 MHz, CD_3OD) spectrum for naamidine J (6)

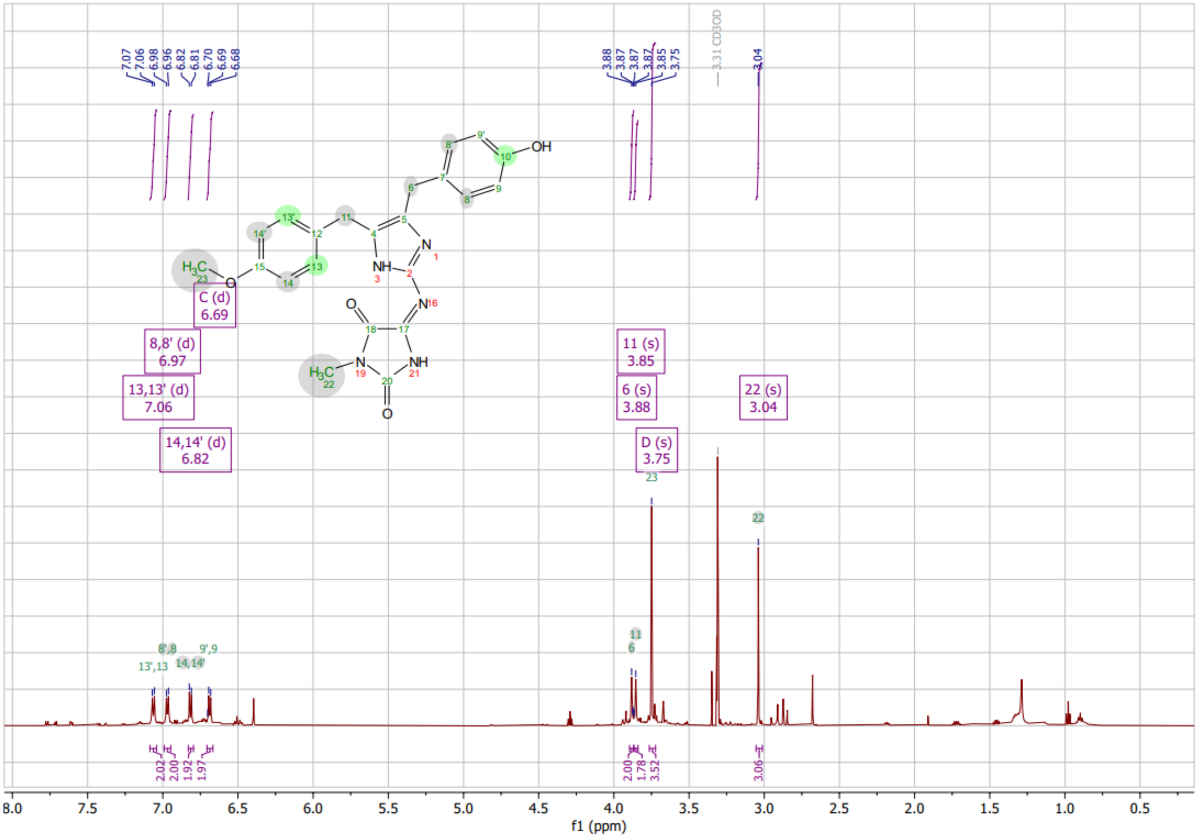

Figure S31: ^{13}C NMR (125 MHz, CD_3OD) spectrum for naamidine J (6)

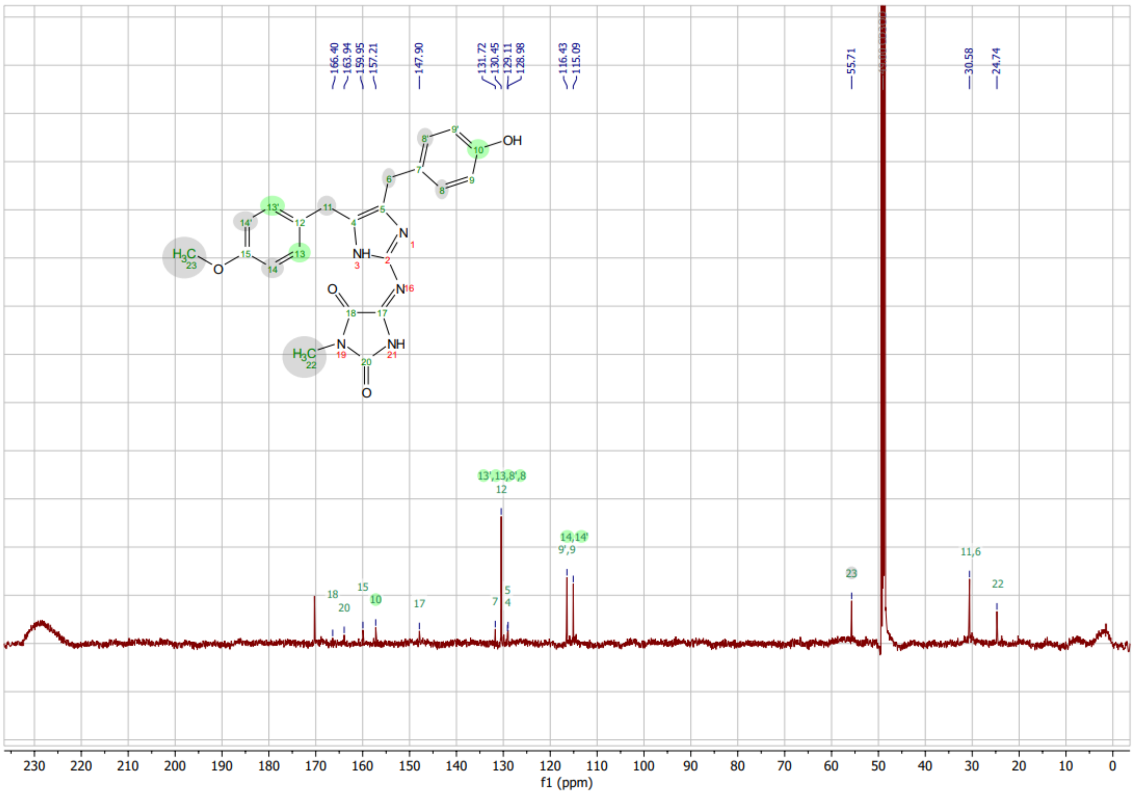

Figure S32: ^{1}H-^{1}H COSY NMR (600 MHz) spectrum for naamidine J (6)

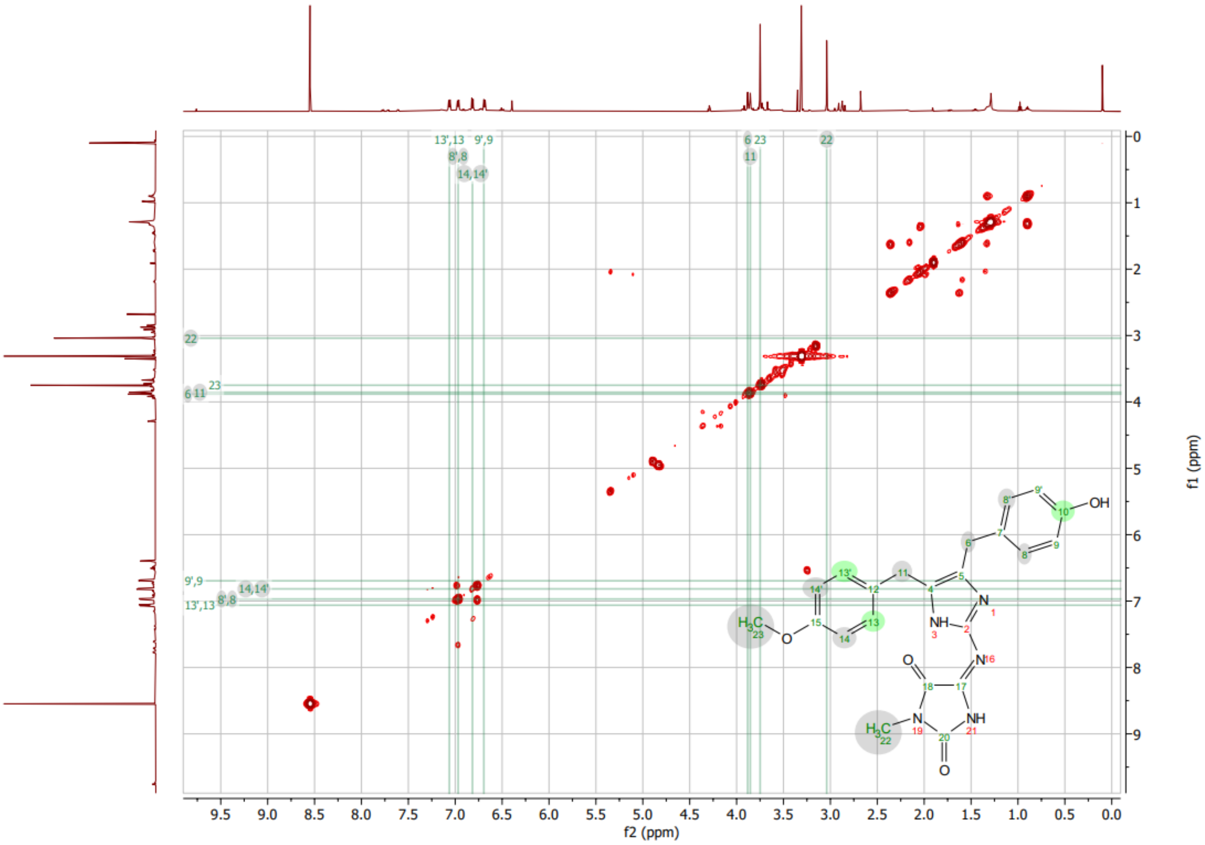

Figure S33: ^{1}H-^{13}C HSQC NMR (600 MHz) spectrum for naamidine J (6)

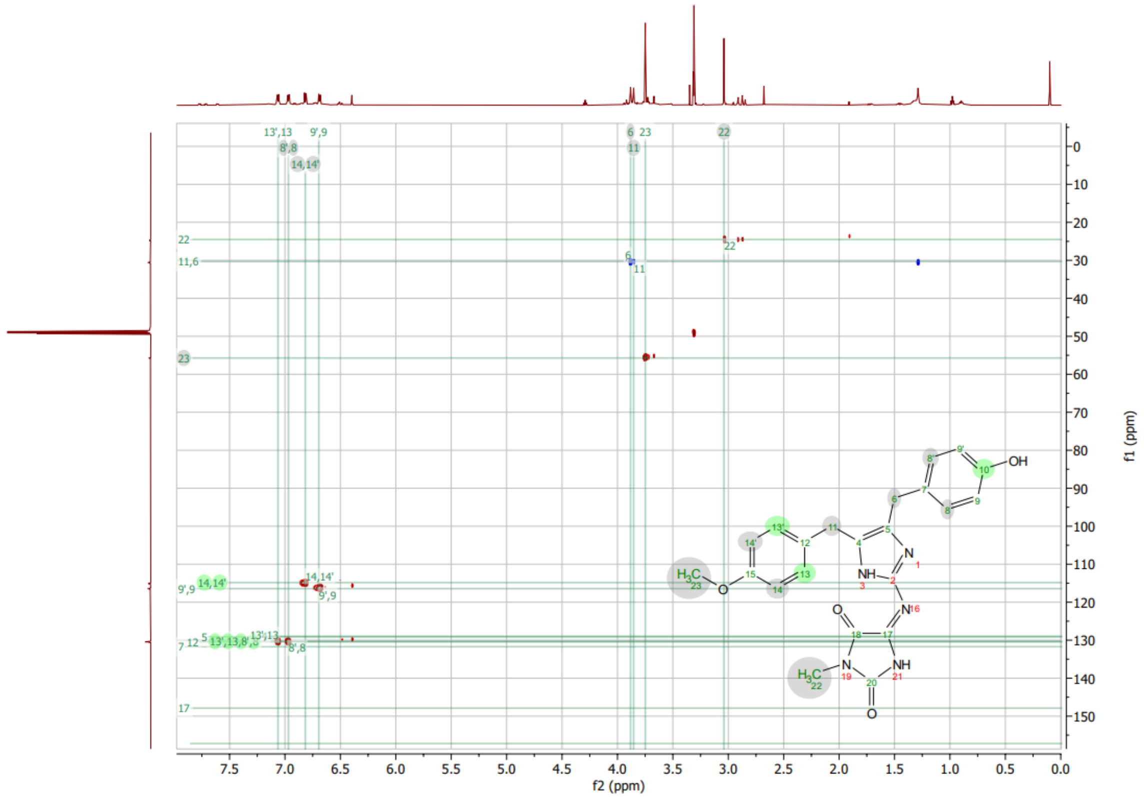

Figure S34: ¹H-¹³C HMBC NMR (600 MHz) spectrum for naamidine J (6)

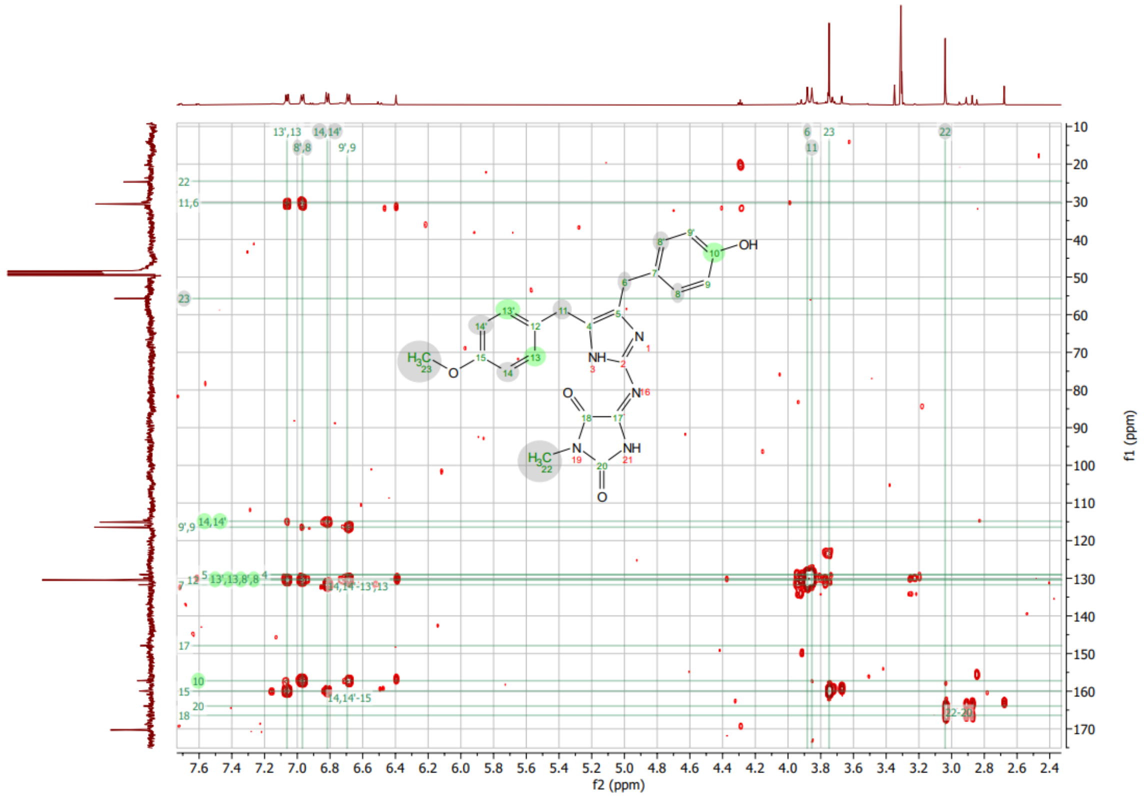

Figure S35: ¹H-¹H NOESY NMR (600 MHz) spectrum for naamidine J (6)

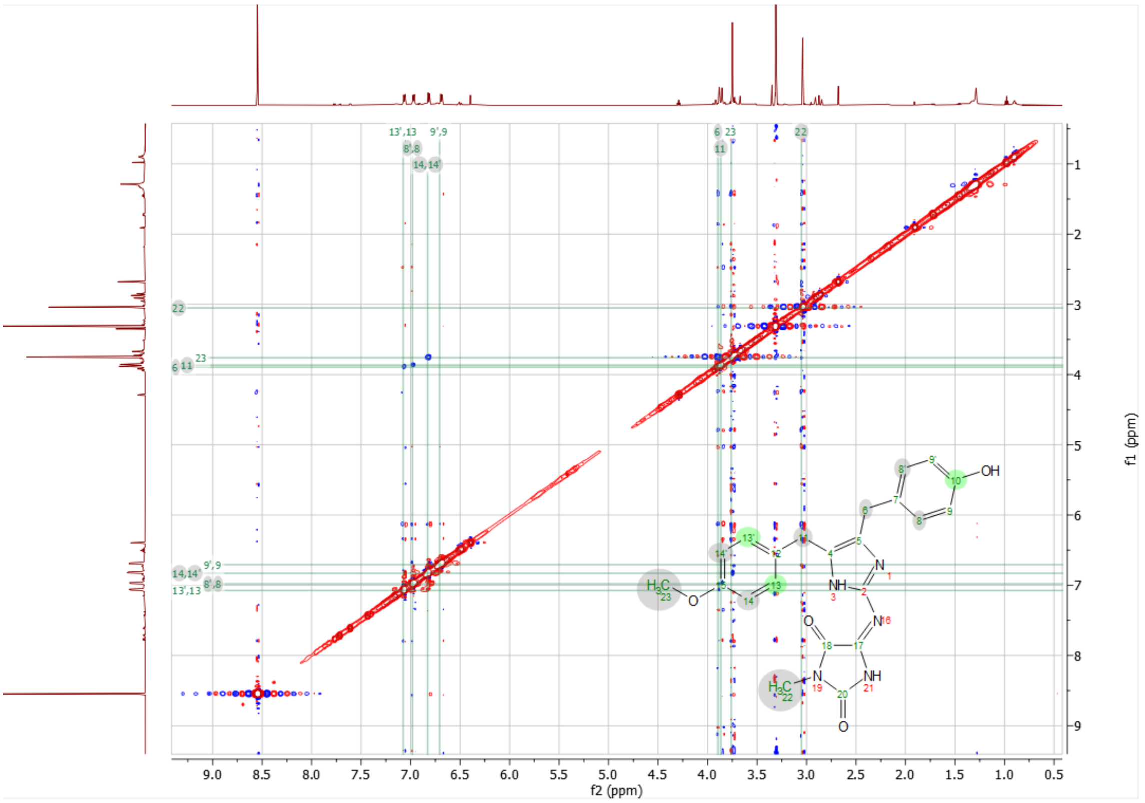

Figure S36: HRESIMS spectrum for naamine I (7)

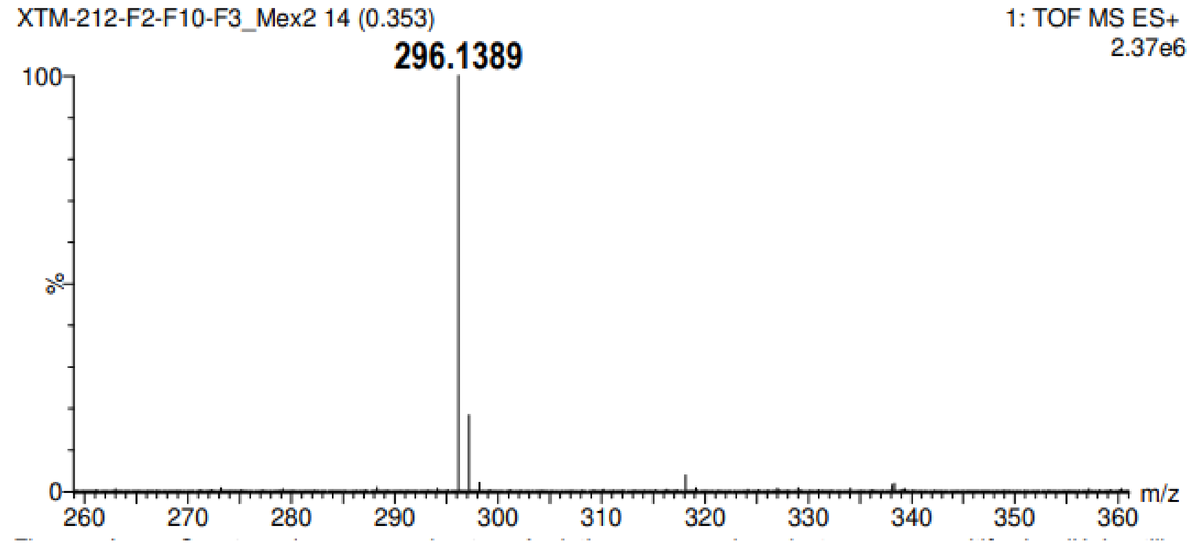

Figure S37: HRESIMS spectrum for naamidine K (8)

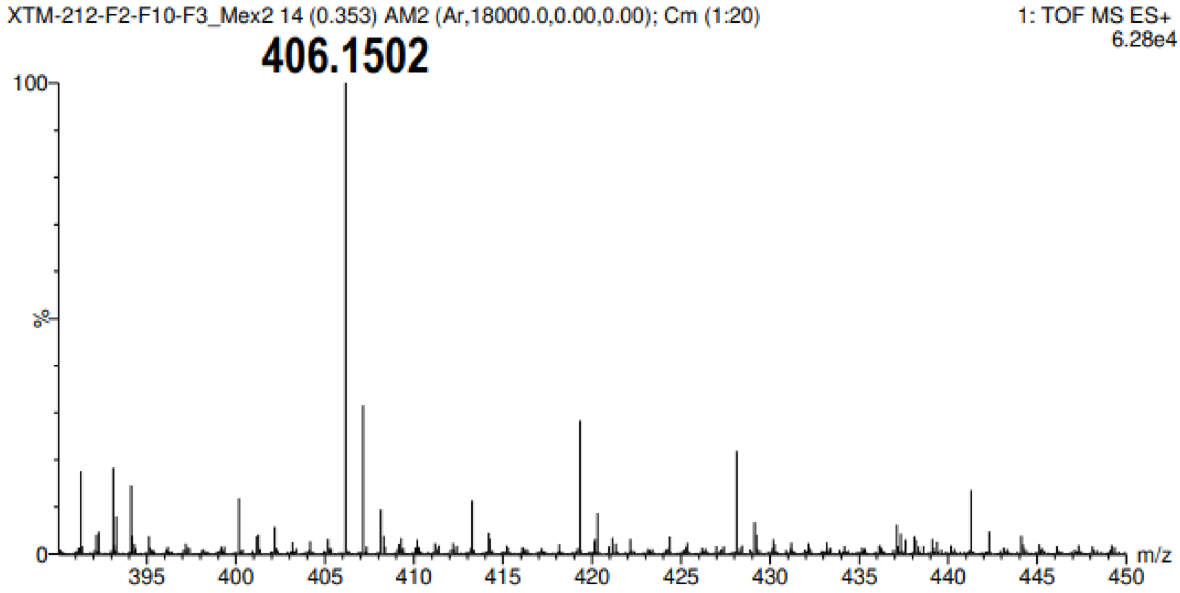

Figure S38: ^{1}H NMR (600 MHz, CD_3OD) spectrum for mixture of naamine I (7) and naamidine K (8)

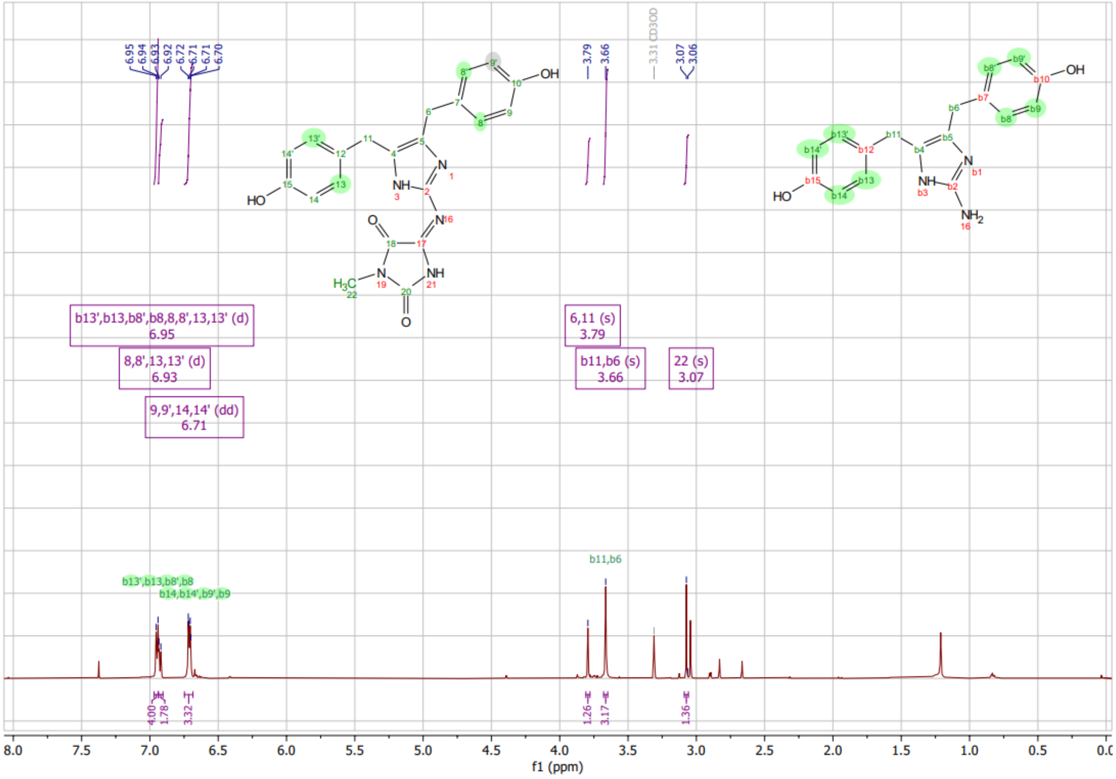

Figure S39: ^{1}H-^{13}C HSQC NMR (600 MHz) spectrum for mixture of naamine I (7) and naamidine K (8)

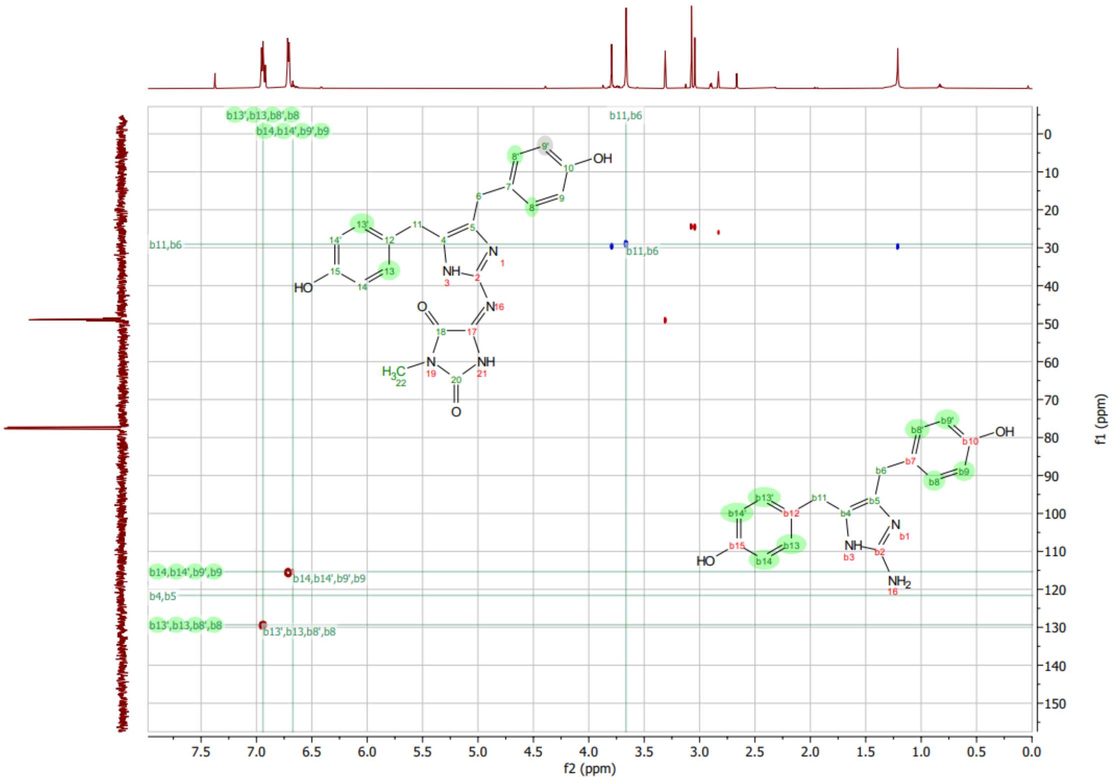

Figure S40: ^{1}H-^{13}C HMBC NMR (600 MHz) spectrum for mixture of naamine I (7) and naamidine K (8)

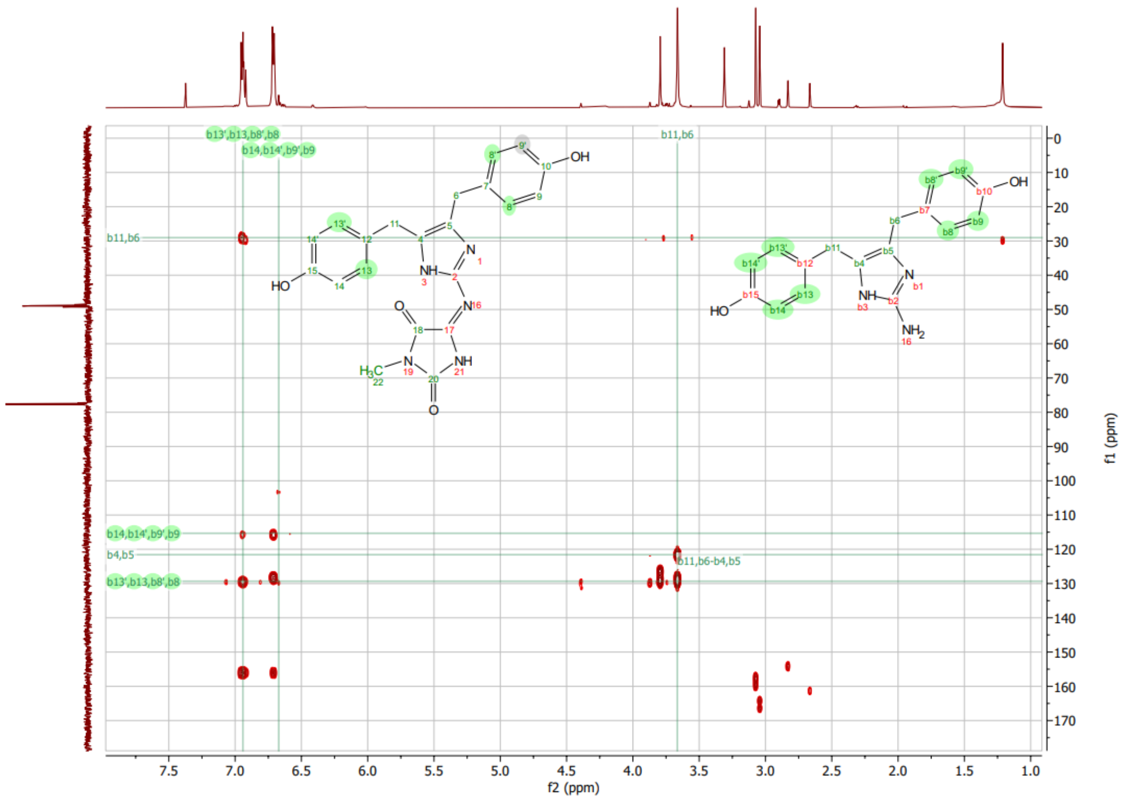

Figure S41: ^{1}H NMR (600 MHz, CD_3OD) spectrum for thymidine (9)

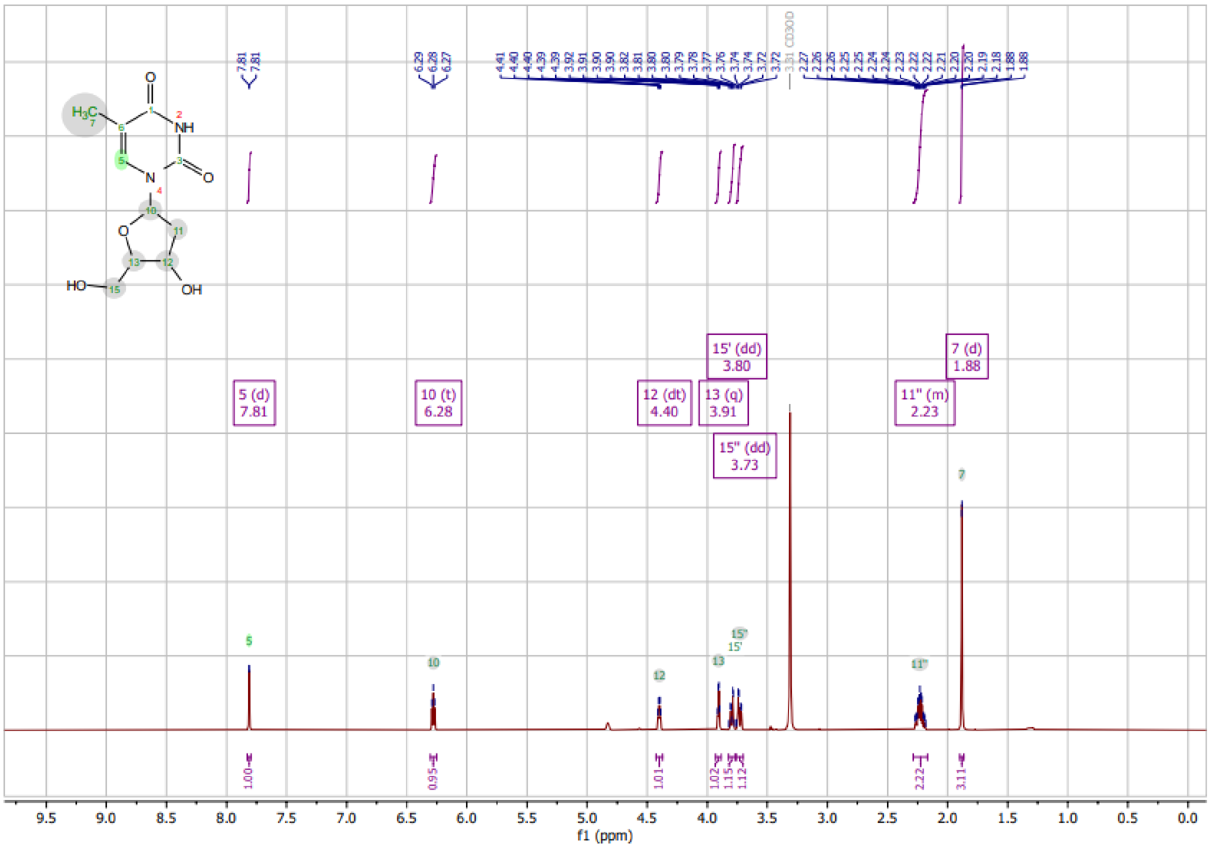

Figure S42: ^{13}C NMR (125 MHz, CD_3OD) spectrum for thymidine (9)

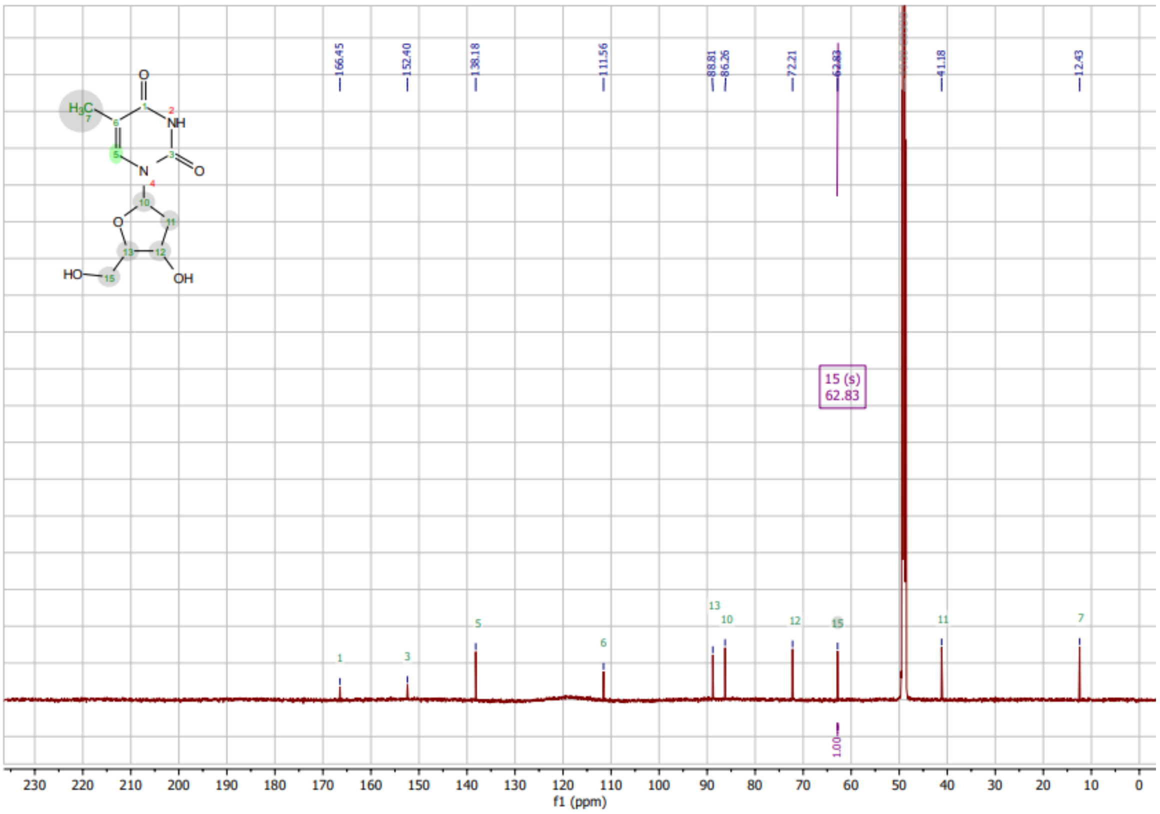

Figure S43: ^1H-^1H COSY NMR (600 MHz) spectrum for thymidine (9)

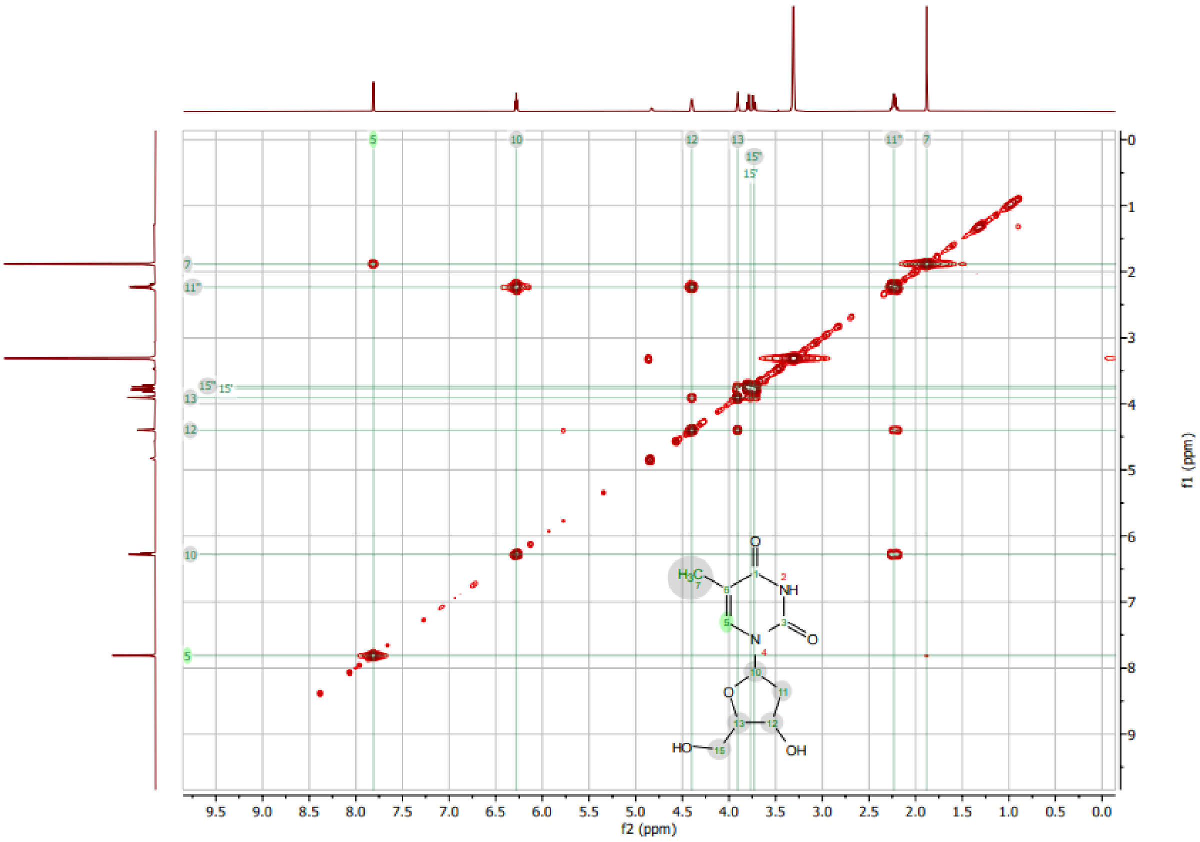

Figure S44: ^{1}H-^{13}C HSQC NMR (600 MHz) spectrum for thymidine (9)

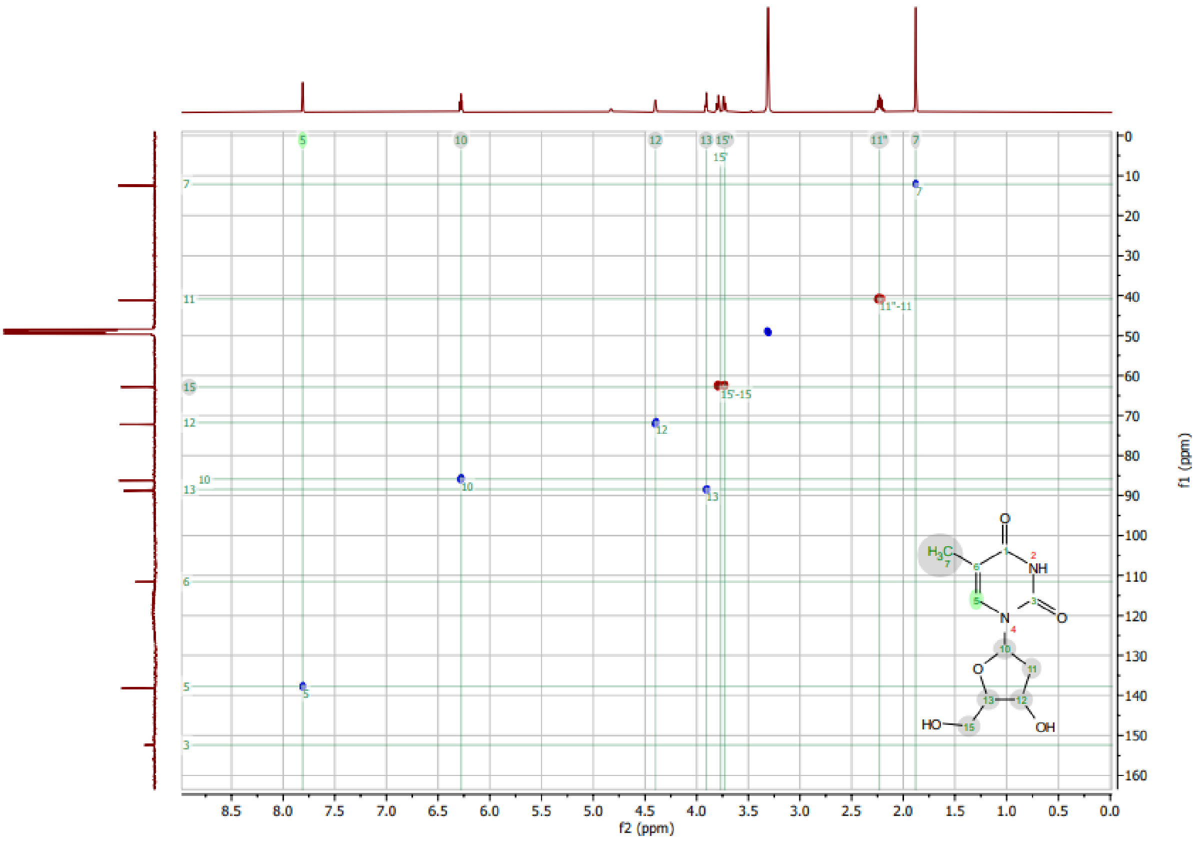

Figure S45: ^{1}H-^{13}C HMBC NMR (600 MHz) spectrum for thymidine (9)

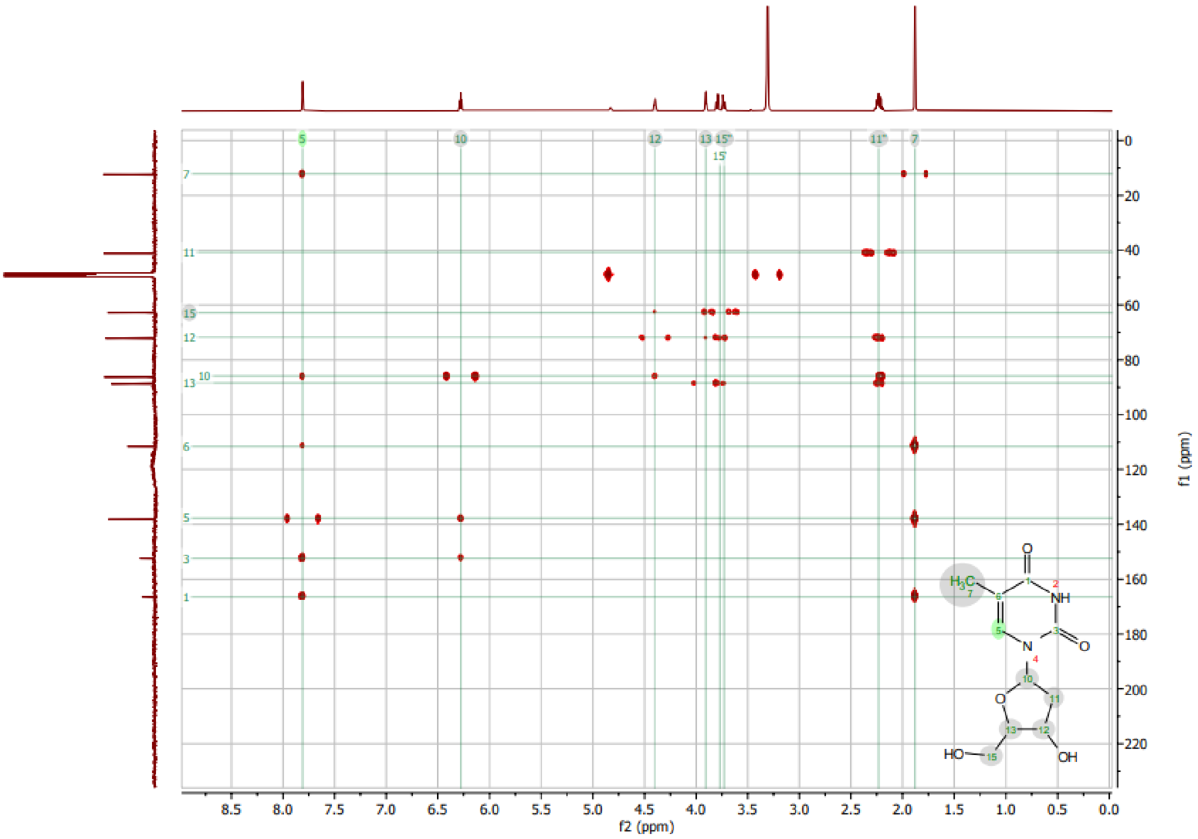

Figure S46: MS/MS spectra of the isolated compounds were deposited in the GNPS spectral libraries under following identifier

Compound	GNPS accession code
Phorbatopin E	CCMSLIB00009919260
Calcaridine C	CCMSLIB00009919261
Erstine A	CCMSLIB00009919262
Naamine H	CCMSLIB00009919263
Naamine I	CCMSLIB00009919264
Naamidine J	CCMSLIB00009919265
Naamidine K	CCMSLIB00009919266

Figure S47: MS/MS spectrum of ernstine A (1)

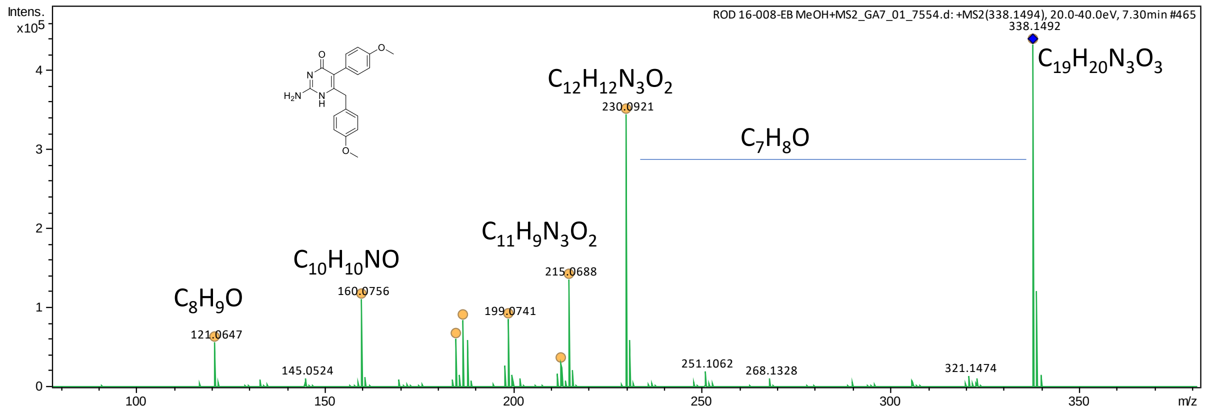

Figure S48: MS/MS spectrum of Phorbatopsin D (2)

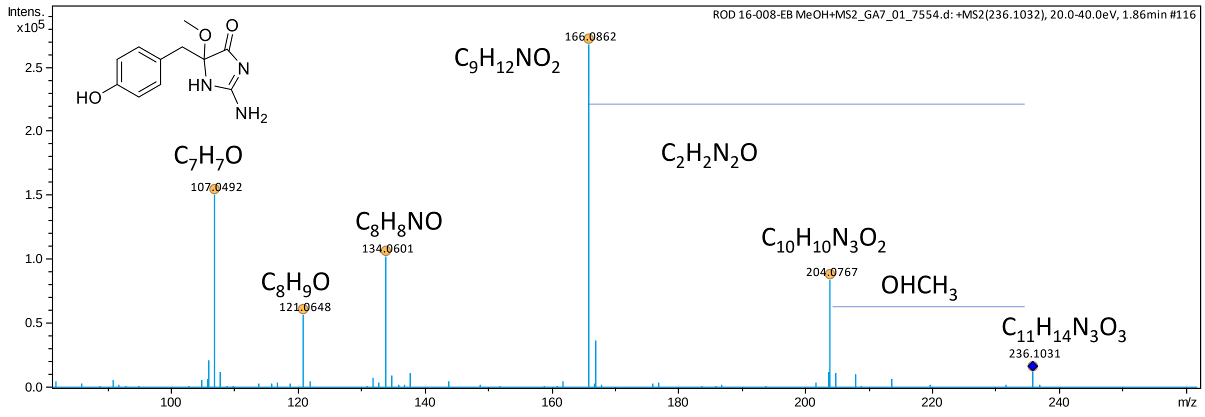

Figure S49: MS/MS spectrum of Phorbatopsin E(3)

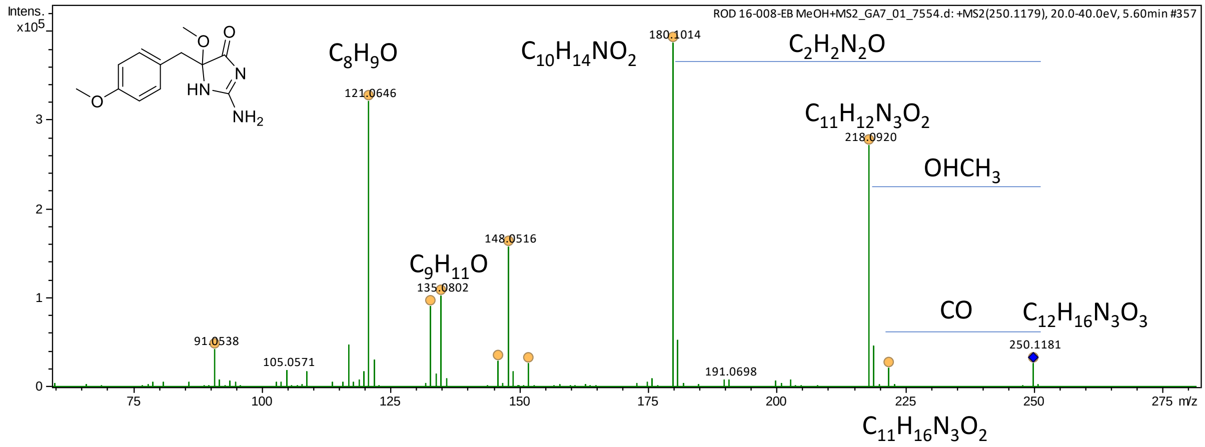

Figure S50: MS/MS spectrum of Naamine H (5)

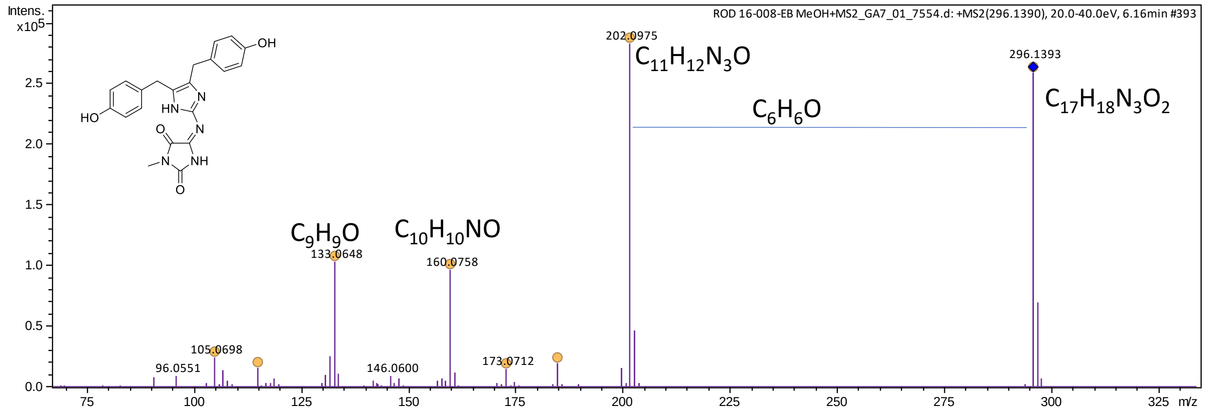

Figure S51: MS/MS spectrum of Naamine I (7)

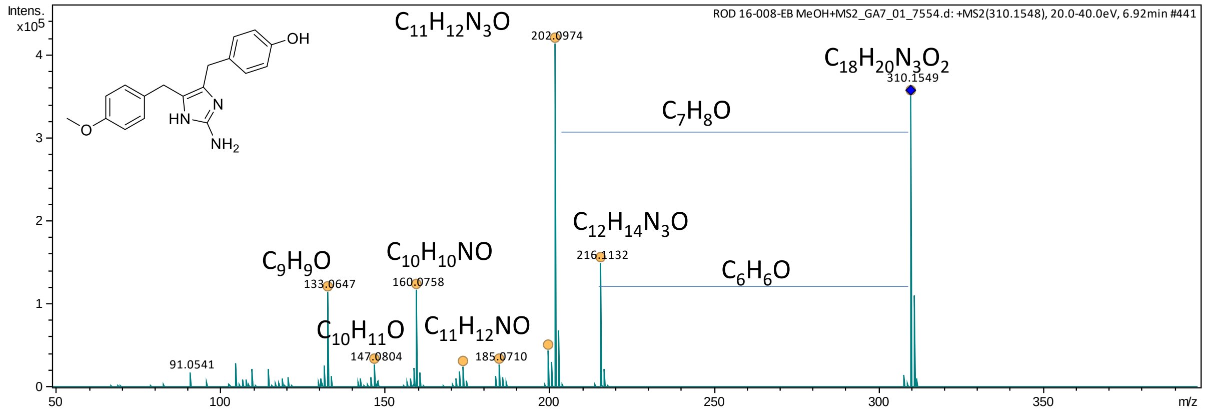

Figure S52: MS/MS spectrum of Naamidine J (6)

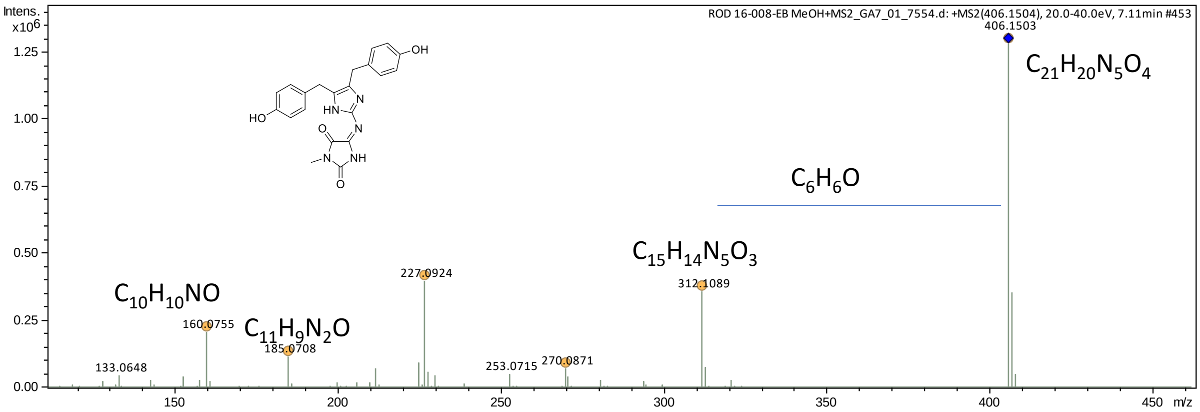

Figure S53: MS/MS spectrum of Naamidine K (8)

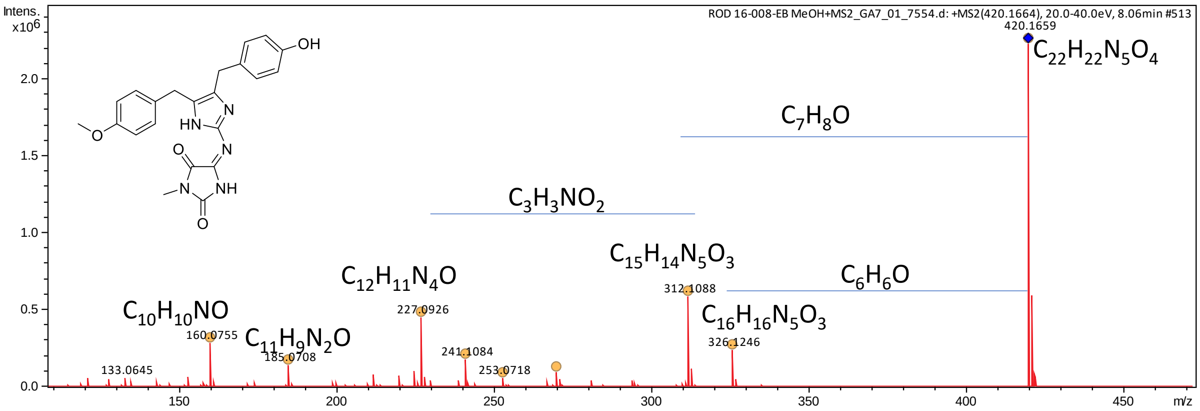

Figure S54: MS/MS spectrum of Calcaridine C (4)

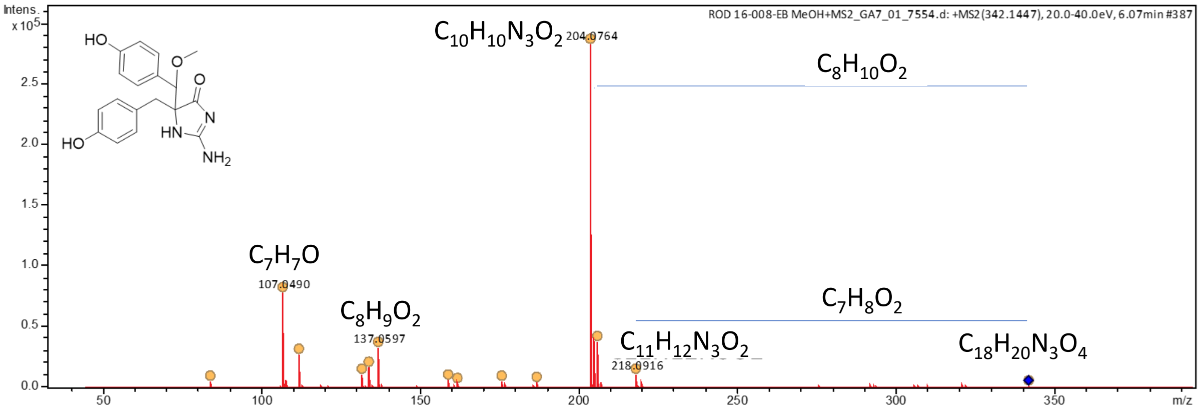

Supplement: Supplementary file 1 [file marinedrugs-20-00637-s001.zip › marinedrugs-1957277-supplementary.pdf]
